# Cost-effectiveness of adjunct non-pharmacological interventions for osteoarthritis of the knee

**DOI:** 10.1371/journal.pone.0172749

**Published:** 2017-03-07

**Authors:** Beth Woods, Andrea Manca, Helen Weatherly, Pedro Saramago, Eleftherios Sideris, Christina Giannopoulou, Stephen Rice, Mark Corbett, Andrew Vickers, Matthew Bowes, Hugh MacPherson, Mark Sculpher

**Affiliations:** 1 Centre for Health Economics, University of York, York, United Kingdom; 2 Centre for Reviews and Dissemination, University of York, York, United Kingdom; 3 Department of Epidemiology and Biostatistics, Memorial Sloan-Kettering Cancer Center, New York, New York, United States of America; 4 York Teaching Hospital NHS Foundation Trust, York, United Kingdom; 5 Department of Health Sciences, University of York, York, United Kingdom; University of Exeter, UNITED KINGDOM

## Abstract

**Background:**

There is limited information on the costs and benefits of alternative adjunct non-pharmacological treatments for knee osteoarthritis and little guidance on which should be prioritised for commissioning within the NHS. This study estimates the costs and benefits of acupuncture, braces, heat treatment, insoles, interferential therapy, laser/light therapy, manual therapy, neuromuscular electrical stimulation, pulsed electrical stimulation, pulsed electromagnetic fields, static magnets and transcutaneous electrical nerve Stimulation (TENS), based on all relevant data, to facilitate a more complete assessment of value.

**Methods:**

Data from 88 randomised controlled trials including 7,507 patients were obtained from a systematic review. The studies reported a wide range of outcomes. These were converted into EQ-5D index values using prediction models, and synthesised using network meta-analysis. Analyses were conducted including firstly all trials and secondly only trials with low risk of selection bias. Resource use was estimated from trials, expert opinion and the literature. A decision analytic model synthesised all evidence to assess interventions over a typical treatment period (constant benefit over eight weeks or linear increase in effect over weeks zero to eight and dissipation over weeks eight to 16).

**Results:**

When all trials are considered, TENS is cost-effective at thresholds of £20–30,000 per QALY with an incremental cost-effectiveness ratio of £2,690 per QALY vs. usual care. When trials with a low risk of selection bias are considered, acupuncture is cost-effective with an incremental cost-effectiveness ratio of £13,502 per QALY vs. TENS. The results of the analysis were sensitive to varying the intensity, with which interventions were delivered, and the magnitude and duration of intervention effects on EQ-5D.

**Conclusions:**

Using the £20,000 per QALY NICE threshold results in TENS being cost-effective if all trials are considered. If only higher quality trials are considered, acupuncture is cost-effective at this threshold, and thresholds down to £14,000 per QALY.

## Introduction

Patients with knee osteoarthritis have a range of treatment options available including pharmacological, non-pharmacological and surgical management. During the last decade emphasis has shifted to non-pharmacological management[1] and it is generally accepted that patients should be offered education, exercise and if appropriate weight management strategies[2]. The role of other adjunct non-pharmacological therapies that can be used alongside these core interventions is less clear.

The only additional non-pharmacological therapies recommended by the European League Against Rheumatism (EULAR) guidelines are the use of appliances (sticks, insoles, knee bracing and other assistive devices) and appropriate footwear[1, 3]. In the UK, the National Institute for Health and Care Excellence (NICE) guideline[2] recommends local heat and cold, manual therapy, transcutaneous electrical nerve stimulation (TENS), braces, joint supports, insoles and assistive devices. Acupuncture was the only non-pharmacological adjunct treatment explicitly not recommended by NICE though a range of other interventions was reviewed. The EULAR recommendations were based on randomised controlled trials (RCTs), observational studies and previous systematic reviews and meta-analyses. NICE recommendations took in to account similar evidence but also reviewed economic evidence and included specifically commissioned meta-analyses and economic analyses.

Decision makers ideally require comparable estimates of the costs and effects of all alternative interventions, based on all relevant evidence, to allow their value to be assessed head-to-head. However, both EULAR and NICE guidance were based on meta-analyses, individual RCTs or cost-effectiveness studies which typically focused on the comparison of two interventions. Using such “pairwise” intervention comparisons to understand the comparative costs and benefits of all available therapies is challenging due to differences in study methods, outcomes and inconsistencies in results.

In addition, both EULAR and NICE guidance were informed by comparisons of interventions using a wide range of patient reported outcomes. Health care decision makers need to make investment decisions across clinical areas and therefore require a common outcome measure. In many jurisdictions, the Quality-Adjusted Life Year (QALY) is used[4]. The QALY reflects an individual’s remaining life expectancy weighted by some measure of health related quality of life (HRQoL). In this context HRQoL is typically measured using an instrument which is relevant across clinical areas and for which data reflecting the general public’s preferences for different HRQoL outcomes is available, such as the EQ-5D[5] measure preferred by NICE[6].

The objective of this study is to assess the cost-effectiveness of a range of adjunct non-pharmacological interventions for use in knee osteoarthritis patients within the UK National Health Service (NHS) using consistent methods to estimate costs and QALYs. Generating comparable estimates that incorporate all relevant evidence for all treatments is challenging. Each available RCT compares a small subset of the available interventions. Furthermore, the EQ-5D is rarely reported, and the HRQoL data that is reported varies. We therefore use network meta-analysis (NMA) and statistical mapping techniques to address these challenges.

## Methods

### Overview

The economic evaluation compares the adjunct non-pharmacological interventions in Table 1 to assess whether any of them represent a cost-effective use of UK NHS resources when used in a general cohort of patients with knee osteoarthritis (age >55 years). The study is conducted from the perspective of the UK NHS and Personal Social Services (PSS). Individuals with osteoarthritis of the knee are usually managed within primary care and may receive these treatments in this context, or via referral (including self-referral) to a musculoskeletal outpatient physiotherapy service. Usual care can be defined as any standard care package which may incorporate regular or intermittent follow-up, self-management strategies, analgesics, education and exercise advice[7]. Given the heterogeneity in what may constitute usual care in practice, the objective of this analysis was to estimate the incremental benefits and costs of the therapies listed in Table 1 over and above those associated with usual care rather than to quantify the outcomes and costs expected under usual care. The interventions appraised are expected to impact on pain and functioning but not on disease progression. Our evaluation therefore focuses on such HRQoL changes as the goal of intervention.

**Table 1:** Interventions evaluated.

Acupuncture	
Appliances	*Braces*
	*Insoles*
Electrotherapy	*Interferential therapy*
	*Laser/light therapy*
	*Neuromuscular electrical stimulation (NMES)*
	*Pulsed electrical stimulation*
	*Pulsed electromagnetic fields*
	*Transcutaneous electrical stimulation (TENS)*
Manual therapy	
Static magnets	
Heat treatment	
Usual care	

The evaluation comprised two components. Firstly a process of statistical mapping and NMA was used to provide comparable estimates of the effect of each intervention on HRQoL measured using the EQ-5D. Statistical mapping techniques[8] were used to translate the variable HRQoL data reported in each RCT to EQ-5D estimates. NMA, an extension of conventional pairwise meta-analysis, was then used to combine evidence from trials comparing different sets of interventions[9–11]. The second component used a decision model to translate these estimates of EQ-5D to QALYs and to estimate costs. Although knee osteoarthritis is a chronic condition, the analysis focuses on the benefit of treatment within a typical treatment period (8 weeks in the UK) as there is limited evidence on the longer-term effects of these interventions.[12] The impact of longer term benefits is explored in sensitivity analyses and returned to in the Discussion. Given the eight week time horizon, no discounting was applied.

### Clinical data

**Fig 1:**
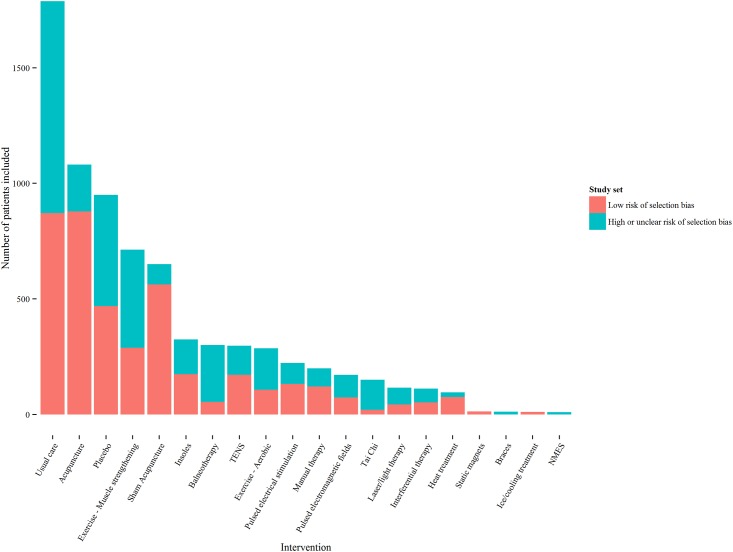
Patients randomised to trials included in the network, by comparator and study quality. NMES = neuromuscular electrical stimulation; TENS = transcutaneous electrical nerve stimulation.

RCTs were identified from a previous systematic review[7]. RCTs were required to have assessed pain as a primary or secondary outcome in adults with knee osteoarthritis and population mean age ≥55 years. The systematic review identified 152 RCTs. For five of the identified studies individual patient data (IPD) were made available from the Acupuncture Trialists’ Collaboration (ATC) repository[13]. IPD are preferred to the data available in published reports as they allow analyses to be tailored to the study question and a consistent analytic approach across studies. Studies were included in the current analysis if reported mean scores for all dimensions of any HRQoL measure listed below (see section ‘Translation of clinical data to EQ-5D estimates’) for one or more post-baseline time points. Studies were included if they reported absolute values, or reported change from baseline alongside baseline data and therefore allowed calculation of absolute values. This allowed 88 studies and 7,507 patients to be included as shown in Fig 1. Follow-up assessments and treatment duration varied across studies. The analysis included data that was reported closest to eight weeks from baseline and whilst patients were on treatment or within two weeks after planned treatment ended. A full list of the included studies and data is provided in the Supplementary Material.

Some interventions were included in the systematic review and NMA but not in the cost-effectiveness analysis. Exercise and weight loss were excluded from the cost-effectiveness analysis as they are core treatments rather than adjunct therapies[2]. Balneotherapy was excluded as it is not used in the UK; ice/cooling treatment was excluded as this is a common self-management strategy with no/minimal cost and no known risk[2] and placebo and sham acupuncture were excluded as it was not expected that either would be prescribed. However all of these interventions were retained in the NMA as they strengthened the network of evidence and provided indirect data to inform HRQOL comparisons of the interventions of interest. Usual care and ‘no intervention’ were pooled as trial reporting did not allow these comparators to be clearly distinguished, and it was expected as all patients included in the trials were diagnosed with osteoarthritis they would be receiving some form of care.

The studies were generally poor quality: only nine (10%) were considered at overall low risk of bias according to the Cochrane risk of bias tool [14]. A previous meta-epidemiologic study of 16 meta-analyses comparing active to control interventions or placebo in patients with hip or knee osteoarthritis found effect sizes to be higher in studies with unclear or inadequate allocation concealment compared to those with adequate allocation concealment according to the Cochrane risk of bias tool [15]. Two different sets of trials were therefore analysed: all 88 trials and 39 trials with low risk of bias for allocation concealment (referred to as trials at low risk of selection bias)[15]. An analysis restricted to trials considered at overall low risk of bias according to the Cochrane risk of bias tool was not possible due to the absence of a connected network of RCTs.

### Translation of clinical data to EQ-5D estimates

The HRQoL instrument(s) collected and reported varied considerably across trials. The EQ-5D was our preferred endpoint to generate QALY estimates given NICE’s preference for the measure.[6] We therefore focused on HRQoL instruments for which a mapping algorithm to EQ-5D was available, identified using a published database[16].

**Fig 2:**
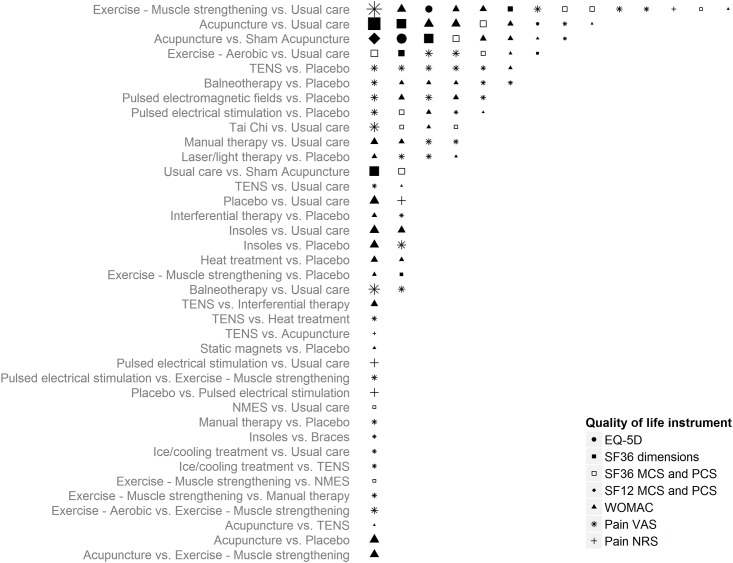
Best available quality of life data by intervention comparison. Each row denotes a pairwise comparison of interventions for which randomised controlled trial data was available. The number of symbols in each row reflects the number of trials making that comparison, shape size is proportional to the size of each study and the type of shape indicates the health related quality of life instrument used. Studies informing multiple comparisons (due to the presence of three or more trial arms) appear for each comparison. MCS = mental component summary score; NMES = neuromuscular electrical stimulation; NRS = numerical rating scale; PCS = physical component summary scores; TENS = transcutaneous electrical nerve stimulation; VAS = visual analogue scale; WOMAC = Western Ontario and McMaster Universities Arthritis Index.

The following hierarchy of HRQoL instruments was used to select data for synthesis (see reference in brackets for mapping algorithm): EQ-5D preference values; SF-36 dimension scores[17]; SF-36 mental and physical component summary[18]; SF-12 mental and physical component summary[19]; Western Ontario and McMaster Universities Arthritis Index total score;[20] pain visual analogue scale[18]; and pain numerical rating scale[21]. Fig 2 summarises the data available for each pairwise comparison. The mapping approaches are detailed in the Supplementary Material.

### Network Meta-Analysis (NMA) of EQ-5D estimates

A NMA of the continuous EQ-5D outcome was conducted on both data sets (all trials and trials at low risk of selection bias). The model extended a previously developed IPD model to include aggregate published data.[22] The model assumed treatment effects were transitive on the absolute EQ-5D scale, that is, the treatment effect for treatment B compared to treatment A (*d*_*AB*_) can be estimated as *d*_*AC*_—*d*_*BC*_[23]. As differences in study designs, populations, and the implementation of interventions seemed likely to generate heterogeneity in the underlying true treatment effects, random effects models were applied. The NMA was conducted using Bayesian methods due to the flexibility these methods afford; full statistical methods are reported in the Supplementary Material. The NMA included all interventions in the systematic review with the exception of weight loss, as including trials restricted to overweight patients was expected to increase heterogeneity within the network.

### Cost data

Intervention costs comprised equipment costs and staff time (see Table 2 and references [24–30]). The costing reflects only the incremental costs associated with interventions. Based on clinical opinion, background usual care costs were expected to apply equally across trial arms, and were therefore omitted. Staff time for delivering interventions was estimated using the weighted average weekly therapist contact time across the included RCTs. This reflects the overall therapist time across multiple sessions, if applicable. Expert opinion from a GP and physiotherapist working with patients with osteoarthritis of the knee indicated that weekly appointments would not be required for braces, heat treatment sleeves, insoles, static magnets or TENS and that instead these would require one 40 minute appointment for prescription and in the case of insoles and braces one further 30 minute follow-up. Because of the UK NHS perspective, costs of delivering acupuncture were based on physiotherapists’ time. For durable equipment (insoles, braces, static magnets) benefits and costs were assumed to be spread across their useable lifetimes. We assumed TENS machines would be used at home for eight weeks and then returned for use by other patients. Costs of consumables, costs per use for machines available at physiotherapy units and any intervention-specific training costs were not included as they were expected to be small.

**Table 2:** Resource use and unit costs (cost year 2012–13).

Intervention	Weekly physiotherapist duration (minutes)^a^						Additional prescription time (minutes)^b^	Equipment included in costing, *assumed lifespan* ^c^
	Data from all trials			Data from trials with low risk of selection bias				
	Weighted average	Min	Max	Weighted average	Min	Max		
Acupuncture	37	18	80	40	20	50	0	None
Braces	0	0	0	0	0	0	70	Brace *0*.*5 years*
Heat treatment–diathermy (73%)^d^	84	60	143	60	60	60	0	None
Heat treatment–sleeve (27%)^d^	0	0	0	0	0	0	40	Sleeve *0*.*5 years*
Insoles	0	0	0	0	0	0	70	Insole *1 year*
Interferential therapy	159	40	245	245	245	245	0	None
Laser/light therapy	105	25	210	60	60	60	0	None
Manual therapy	63	30	90	57	30	90	0	None
NMES	100	100	100	NA^e^	NA^e^	NA^e^	0	None
Pulsed electrical stimulation	82	57	114	85	57	114	0	None
Pulsed electromagnetic fields	303	80	600	120	120	120	0	None
Static magnets	0	0	0	0	0	0	40	Magnet *2 years* +strap^f^
TENS	0	0	0	0	0	0	40	TENS machine *1 year*
Source:	Pooled randomised controlled trial data (see text for full source)						Clinical opinion	Clinical opinion

NA = not available (treatment does not provide data to inform network); NMES = neuromuscular electrical stimulation; TENS = transcutaneous electrical nerve stimulation.

^a^Unit costs: £36 (hospital physiotherapist, per hour).

^b^Prescription and follow-up were assumed to be undertaken by a physiotherapist, with the exception of insole prescription and fitting which was assumed to be carried out by a podiatrist (unit cost £30 for community podiatrist, per hour).

^c^Unit costs were £88 (Bauerfeind GenuTrain Knee Support brace); £11 (Titanium adjustable knee-heating strap); £50 (Ready-made lateral wedge foot insole); £50 (Bioflow magnet and separate strap); £35 (TENS digital pain relief unit).

^d^Heat treatment included trials of diathermy and one trial of a heat retaining sleeve. Their costs were therefore weighted according to the proportion of patients in the trials.

^e^No trials of NMES included in this analysis;

^f^50% of patients assumed to require a replacement strap during two years of use. Note: Resource use from the Topical or Oral IBuprofen for chronic knee pain in older people trial was costed as follows: £45 (GP, per visit), £135 (secondary care specialist, per visit).

Interventions which improve symptoms may have indirect effects on health care utilisation. None of the included RCTs reported relevant healthcare utilisation data for the UK. Therefore, data from the Topical or Oral IBuprofen for chronic knee pain in older people (TOIB) trial[30] were used to estimate the extent to which changes in the EQ-5D yield changes in resource utilisation. We assumed that information on the relationship between EQ-5D and resource utilisation from this pharmacological trial was generalizable to the current evaluation of non-pharmacological therapies. A simple ordinary least squares regression estimated that a 0.10 improvement in the EQ-5D between months three and 12 resulted in a 0.09 (95% CI: -0.02, 0.19) reduction in the number of primary care visits from months zero to three to months three to 12 and a 0.05 (95% CI: -0.06, 0.16) increase in specialist visits from months zero to three to months three to 12. This analysis allows for an assessment of whether changes in quality of life between months three and 12 are associated with changes in resource use between months zero to three to months three to 12. The results may reflect a lack of effect as the confidence intervals were wide and included zero. We assumed that this relationship between EQ-5D and resource use could be applied to the model time horizon of eight weeks. For example, an intervention that improved quality of life by 0.10 compared to usual care would result in a 0.09 reduction in primary care visits and a 0.05 increase in specialist visits over the eight week period (as any changes in usual care (baseline) EQ-5D cancel out).

### Cost-effectiveness analysis

#### Decision analytic model

The decision analytic model translated EQ-5D estimates to QALY estimates using the area under the curve method[4]. EQ-5D estimates were assumed to apply for the eight-week time horizon in order to calculate the area under the curve. This approach captures different profiles of therapeutic effect. For example, some interventions (e.g. TENS) may provide rapid relief but confer no residual effect beyond treatment, whereas others (e.g. acupuncture) may require time to deliver full therapeutic effect but effects may dissipate more gradually following cessation. In the latter case if there is a linear increase in effect between baseline and week eight followed by a linear decrease between weeks eight and 16 this will result in the same area under the EQ-5D curve (i.e. QALY estimate) as assuming an instantaneous and constant benefit which is lost at week eight.

Incremental cost-effectiveness results are presented to allow simultaneous comparison of all treatments.[4] In each analysis, the most effective intervention with an incremental cost-effectiveness ratio (ICER) that is less than the cost-effectiveness threshold is the cost-effective choice. The cost-effectiveness threshold represents the maximum the NHS should be willing to spend to generate additional QALYs, in the UK values of £20,000–30,000/QALY[6] are typically used.

#### Sensitivity analyses

Probabilistic sensitivity analysis was conducted to calculate the probability that each intervention is cost-effective and to estimate the value of resolving all uncertainty (the value of perfect information). The posterior distribution from the NMA was used to reflect uncertainty in treatment impacts on EQ-5D, and the uncertainty in the relationship between EQ-5D and primary care/outpatient resource use was represented using a normal distribution.

Scenario analyses explored using the shortest/longest weekly therapist time across trials to determine costs (for braces, insoles, static magnets and TENS, where cost was driven by equipment as well as staff costs, total costs were varied by +/-50%); use of upper and lower 95% credible intervals for effectiveness; and use of weekly time spent with a therapist that is more typical of clinical practice within the NHS (20 minutes, or 30 minutes for acupuncture and manual therapy). The latter analysis is combined with a series of assumptions about how the weekly time spent with a therapist may affect therapeutic benefit: (i) outcomes increase linearly with time spent with a therapist; (ii) outcomes increase linearly to a maximum at one hour; (iii) 75% of outcomes are achieved within 30 minutes and the remaining 25% with the extension to one hour; and (iv) full benefit is achieved within 20–30 minutes.

Two analyses explored the sensitivity of the model results to the possibility that interventions offer longer-term HRQoL gains. Firstly, a threshold analysis was conducted to identify the extension to the duration of benefit required to alter the cost-effective intervention when the benefit of all interventions was extended simultaneously. Secondly, an analysis was conducted to see the impact of extending the benefit of each intervention individually up to a maximum of 50% additional benefit, this reflected feedback from clinical experts that impacts on HRQoL are commonly short-lived and that a linear decline over eight weeks (i.e. 50% additional benefit) may represent a typical maximum on the possible long-term effects of interventions.

## Results

### Effect of interventions on EQ-5D

**Fig 3:**
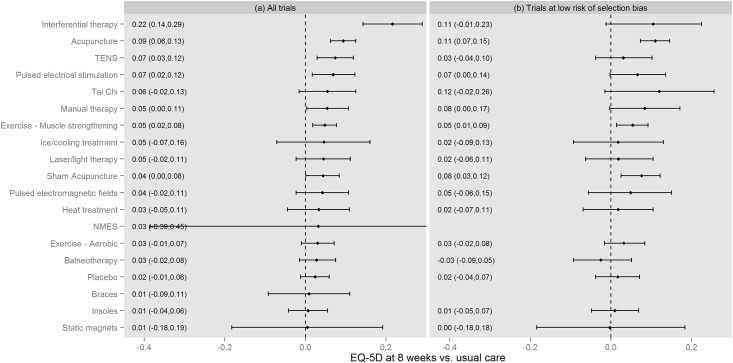
Results of network meta-analyses of EQ-5D. Results presented as values corresponding to 2.5%, 50% and 97.5% of the posterior distribution. NMES = neuromuscular electrical stimulation; TENS = transcutaneous electrical nerve stimulation.

Fig 3 presents the results of the NMA. The level of uncertainty regarding the effect of some comparators was very high, particularly for NMES and static magnets. The 95% credible intervals cross zero with the exception of: muscle strengthening exercise and acupuncture (in both analyses); interferential therapy, pulsed electrical stimulation and TENS (in the all-trials analysis only); and sham acupuncture (in the low risk of selection bias analysis only). The effect of TENS is smaller and the effect of Tai Chi, sham acupuncture and manual therapy larger when the analysis is restricted to trials with low risk of selection bias.

Global tests of model fit suggested an adequate fit to the data. However, the model was unable to fit well to four data points in the all-trials analysis. Two of the data points related to the trials including interferential therapy as a comparator, these trials all suggest that interferential therapy is beneficial but provide inconsistent information regarding effect size[31–33]. The two other data points were from a study comparing aerobic exercise to usual care which has previously been identified as an outlier[12].

### Cost-effectiveness of alternative interventions

**Fig 4:**
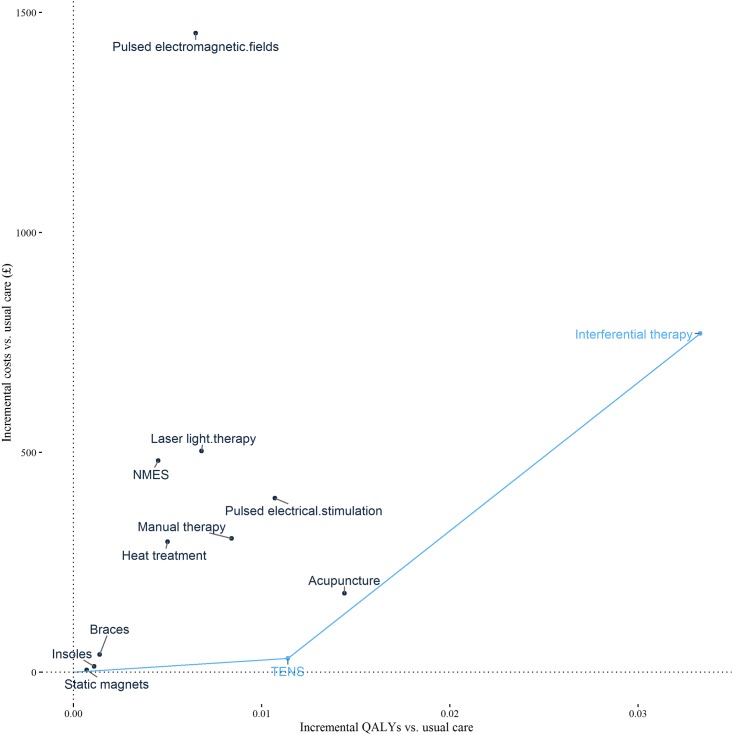
Cost-effectiveness plane including cost-effectiveness frontier: All trials. Each point denotes a comparator and the line denotes the cost-effectiveness frontier. This links all non-dominated comparators and therefore shows the set of comparators that could be cost-effective depending upon the cost-effectiveness threshold. The slope of the line connecting a comparator on the cost-effectiveness frontier to a lower cost comparator is equal to the incremental cost-effectiveness ratio (ICER). NMES = neuromuscular electrical stimulation; TENS = transcutaneous electrical nerve stimulation.

**Fig 5:**
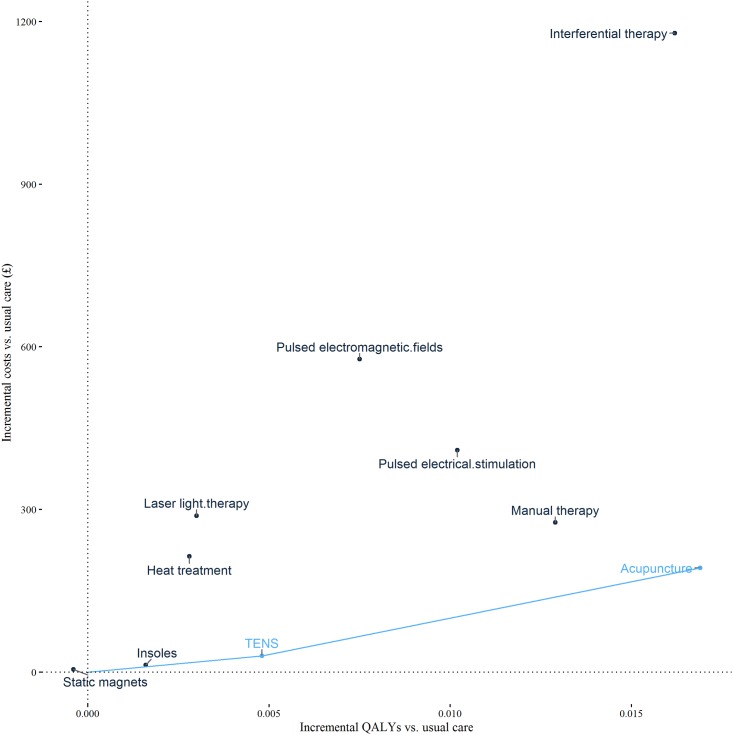
Cost-effectiveness plane including cost-effectiveness frontier: Trials at low risk of selection bias. Each point denotes a comparator and the line denotes the cost-effectiveness frontier. This links all non-dominated comparators and therefore shows the set of comparators that could be cost-effective depending upon the cost-effectiveness threshold. The slope of the line connecting a comparator on the cost-effectiveness frontier to a lower cost comparator is equal to the incremental cost-effectiveness ratio (ICER). TENS = transcutaneous electrical nerve stimulation.

Results of the cost-effectiveness analysis are presented as Table 3 and Fig 4 for all trials and Fig 5 for trials with low risk of selection bias. For some options ICERs are not presented because the intervention is either dominated (generates fewer QALYs and higher costs than another intervention) or because the intervention is extendedly dominated (generates fewer QALYs and has a higher ICER than another intervention). Multiple ICERs show the value of moving to successively more effective interventions. In the analysis of all trials there are two ICERs. The first ICER is the estimated cost per QALY of moving from usual care to TENS. At £2,690 per QALY, this ICER is below the threshold of £20,000–30,000 per QALY and would therefore be considered to represent value for money. The cost per QALY of moving from TENS to interferential therapy is £33,866 per QALY. This ICER exceeds the threshold range considered to represent value for money. In the all trials analysis TENS is therefore cost-effective. In the analysis of trials with low risk of selection bias, the move from usual care to TENS generates value with an ICER of £6,142 per QALY, and the move from TENS to acupuncture generates further value with an ICER of £13,502 per QALY. Acupuncture is therefore cost-effective in this analysis of trials with a low risk of selection bias.

**Table 3:** Cost effectiveness results.

Intervention	All trials			Trials at low risk of selection bias		
	Incremental costs (vs. usual care)	Incremental QALYs (vs. usual care)	ICER (£/QALY)*	Incremental costs (vs. usual care)	Incremental QALYs (vs. usual care)	ICER (£/QALY)*
Static magnets	£5	0.001	ED	£5	0.000	Dom
Insoles	£13	0.001	ED	£13	0.002	ED
TENS	£31	0.011	**£2,690**	£30	0.005	£6,142
Braces	£40	0.001	Dom	NA	NA	NA
Acupuncture	£179	0.014	ED	£192	0.017	**£13,502**
Heat treatment	£297	0.005	Dom	£214	0.003	Dom
Manual therapy	£304	0.008	Dom	£276	0.013	Dom
Pulsed electrical stimulation	£396	0.011	Dom	£410	0.010	Dom
NMES	£481	0.005	Dom	NA	NA	NA
Laser light therapy	£503	0.007	Dom	£288	0.003	Dom
Interferential therapy	£770	0.033	£33,866	£1,179	0.016	Dom
Pulsed electromagnetic fields	£1,453	0.007	Dom	£577	0.008	Dom

Dom: Dominated (generates fewer QALYs and equal/higher costs than another intervention); ED = Extendedly dominated (generates fewer QALYs and has a higher incremental cost-effectiveness ratio than another intervention); NA = not available as no trials of this therapy were available in the analysis.

^a^ Each ICER is calculated as the incremental cost per QALY of the intervention compared to the next less effective intervention which is not dominated or extendedly dominated. The cost-effective intervention is the most effective intervention which still represents value for money, in the UK ICERs less than £20–30,000 per QALY are generally considered to represent value for money. The ICER associated with the cost-effective intervention is in bold.

There is a 49% probability that TENS is cost-effective in the all trials analysis (at a cost-effectiveness threshold of £20,000/QALY) and a 47% probability that acupuncture is cost-effective in the analysis of trials with low risk of selection bias. The net health benefit of resolving all uncertainty is 60% greater than the health achieved by making decisions based on current information for the analysis of all trials; and 50% higher for the analysis of trials at low risk of selection bias.

The majority of scenario analyses did not alter the cost-effective interventions. Table 4 shows the subset of analyses in which the decision was altered. The results of the analysis were sensitive to varying weekly time spent with a therapist, effects of interventions on HRQoL, and to how HRQoL evolves over time.

**Table 4:** Results of sensitivity analyses which altered cost-effective intervention.

Dataset	Scenario	The intervention that is cost-effective for each scenario at £20,000/QALY
**All trials**	Base case	TENS
	Shortest weekly therapist time used for acupuncture costing	Acupuncture
	Shortest weekly therapist time used for interferential therapy costing	Interferential therapy
	Shortened weekly therapist time—75% of benefit in first 30 mins, remainder by 1 hour	Interferential therapy
	Shortened weekly therapist time—all benefit achieved within 20–30 minutes	Interferential therapy
	Increase in duration of benefit of all interventions by 6 weeks	Interferential therapy
	Increase in duration of benefit of acupuncture by 31%	Acupuncture
	Increase in duration of benefit of interferential therapy by 45%	Interferential therapy
	Lower 95% CrI from NMA for TENS	Acupuncture
	Upper 95% CrI from NMA for Acupuncture	Acupuncture
	Upper 95% CrI from NMA for Braces	Braces
	Upper 95% CrI from NMA for NMES	NMES
	Upper 95% CrI from NMA for Static magnets	Static magnets
**Trials at low risk of selection bias**	Base case	Acupuncture
	Shortened weekly therapist time—all benefit achieved within 20–30 minutes	Interferential therapy
	Lower 95% CrI from NMA for Acupuncture	TENS
	Upper 95% CrI from NMA for Insoles	Insoles
	Upper 95% CrI from NMA for Manual therapy	Manual therapy
	Upper 95% CrI from NMA for Static magnets	Static magnets
	Upper 95% CrI from NMA for TENS	TENS

NMA = network meta-analysis; NMES = neuromuscular electrical stimulation; TENS = transcutaneous electrical nerve stimulation; CrI = credible interval.

## Discussion

This is the first analysis to provide comparable estimates of costs and QALYs for the range of adjunct non-pharmacological treatments for knee osteoarthritis, based on all relevant RCT data.

There is a difference between analyses with respect to whether TENS or acupuncture is the cost-effective treatment. The all-trials analysis indicates TENS is cost-effective whereas the analysis restricted to trials at low risk of selection bias indicates that acupuncture is cost-effective. This difference is driven by a reduction in the TENS treatment effect in the latter analysis. This suggests that the effect of TENS may be exaggerated in some trials included in the all trials analysis due to biases associated with poor trial conduct.

There is considerable uncertainty around the effects of interventions—though less so for acupuncture and muscle-strengthening exercise (as shown in Fig 3) where a relatively large number of patients informed both the analysis of all trials and the analysis restricted to trials at low risk of selection bias (as shown in Fig 1). There is also considerable uncertainty in the probability that each intervention is cost-effective. This reflects uncertainty in effect sizes for most of the interventions and the large number of interventions. However, decisions regarding adoption of interventions should not be based on the probability that an intervention is cost-effective, and instead should reflect the costs and benefits of alternative policy options available to decision makers[34]. Decision makers can decide to adopt an intervention without further research, adopt the intervention alongside research or delay adoption until further research is available[35]. Delaying adoption may be preferable if immediate adoption would impede valuable research (for example adoption may remove physicians’ or patients’ incentives to participate in research). However, delaying adoption also has a cost as the benefits of adopting TENS or acupuncture during the research period are foregone. In the current analysis decisions made with perfect information would generate 50%-60% more health than decisions made with current information. Given that research will take a number of years to report, only partially resolve uncertainty, and incur research costs, it is unlikely that delaying adoption until further research is available would be beneficial. A further benefit of delayed adoption is that it avoids upfront investment costs which may turn out to have been unwise if research results in a reversal of the decision. We do not anticipate upfront costs of sufficient magnitude to make delaying adoption the preferred policy. Adoption, with or without further research is therefore the preferred policy.

The recent NICE osteoarthritis guidelines included recommendations regarding the use of various non-pharmacological adjunct interventions, though they only reviewed economic evidence relating to acupuncture [2]. The NICE Guideline Development Group concluded that although acupuncture was likely to be cost-effective, it should not be recommended given a lack of clear clinical benefit over and above sham acupuncture. Our analysis assumes that sham acupuncture would not be prescribed and that acupuncture should therefore be compared to other viable adjunct interventions (including no adjunct therapy i.e. usual care). Our analysis also includes all alternative interventions and by doing so finds that some interventions recommended by NICE—namely insoles, braces and manual therapy—are unlikely to be cost-effective and should not be prioritised for commissioning.

The conclusions of the current work are based on a cost-effectiveness threshold of £20–30,000 per QALY as this has been historically used by NICE. A cost-effectiveness threshold is used to assess whether the health benefits offered by an intervention are greater than the health likely to be lost because the additional resources required are not available to fund other effective treatments. Research conducted during the course of this study suggests that cost-effectiveness thresholds of £20–30,000 per QALY may be too high as it estimated that £13,000 of NHS resources adds one QALY to NHS patients [36]. In this study, using this lower estimate of the cost-effectiveness threshold would result in TENS being the cost-effective choice in both analyses.

This study has a number of limitations. The underlying quality of the RCTs was generally poor. This was addressed in this study by performing an analysis of trials at low risk of selection bias, as this aspect of study quality has been previously found to be a strong marker for effect bias in osteoarthritis.[15] Nonetheless, this represents only one source of bias, and within the studies at low risk of selection bias a number had a high overall risk of bias due to imbalances in baseline characteristics (often caused by small study sizes), a lack of adequate blinding and a failure to report intention-to-treat results.[7] Unfortunately restricting the analysis to only those trials at low risk of bias across domains was not feasible due to the low number of studies that remained. An alternative approach to addressing study bias is to attempt to adjust for it [37]. In the current context an adjustment approach could be applied to address residual biases in the studies at low risk of selection bias, or to synthesise all trials whilst adjusting for biases. However, this type of analysis requires a clear understanding of where biases may be observed. In comparisons with usual care it may be reasonable to assume the biases act in favour of active therapy; however in active treatment comparisons it is difficult to predict the direction of possible biases. In this context the use of elicitation techniques to quantify study-specific biases may be the most fruitful approach though would be highly resource intensive given the number of studies.[37]

The analysis focused on the short-term benefits of treatment as there were insufficient data to provide robust estimates of how HRQoL evolves over time. In an earlier report of the systematic review underpinning this work, the study authors found that only 23% of those studies reporting data suitable for synthesis reported data between eight and 16 weeks from the end of treatment. The available evidence did not form a connected network suitable for analysis, and much of the evidence was from exercise-related trials and was therefore not directly relevant to the decision problem considered here. Recent evidence from an analysis of trials in a wider set of chronic pain conditions (including musculoskeletal pain and headache/migraine as well as osteoarthritis of the knee) estimated that 90% of the benefit of acupuncture over usual care is retained at 12 months after the end of a course of treatment.[38] It is plausible that the other interventions appraised in this study could also continue to directly provide symptomatic benefits beyond the treatment period or, by improving symptoms, could allow individuals to better engage with self-management strategies to improve muscle strength and functioning. We conducted sensitivity analyses examining the impact of extending the duration of all intervention jointly and in turn. This analysis showed that the cost-effective adjunct intervention was sensitive to extending the duration of all therapies, and the duration of acupuncture and interferential therapy individually in the analysis of all trials. Though this shows that duration of therapeutic effect is an important determinant of cost-effectiveness, to establish the implications of this for decision making, an improved understanding of the long-term effects of all interventions is required. Further research is required to explore the long-term benefits of the appraised interventions.

The RCT dataset was heterogeneous. The time point of data collection ranged from one day to one year, although an analysis restricting the set of trials to those reporting within three-13 weeks did not alter the study results (for further details see MacPherson et al.[39]). There were variations in the protocol for care, as well as the intensity with which interventions were provided which may influence outcomes[40], and warrants further exploration. The RCT dataset included studies conducted in a wide range of countries. Differences in the management of chronic pain may therefore have contributed to heterogeneity. The variety in HRQoL measures used in the RCTs necessitated a mapping approach to convert these outcomes to EQ-5D. Mapping is always a second-best approach compared to directly collecting data on preference based measures such as the EQ-5D [8]. None of the RCTs provided resource use data that could be used within the evaluation. We therefore assumed that information on the relationship between EQ-5D and resource utilisation from a pharmacological trial (the TOIB trial) was generalizable to the current evaluation of non-pharmacological therapies, this assumption increases uncertainty around the differences across interventions in costs.

## Conclusions

Using the £20–30,000 per QALY NICE threshold (and any threshold up to about £34,000 per QALY) results in TENS being cost-effective if all trials are considered. If only higher quality trials are considered, acupuncture is cost-effective at the NICE threshold, at any higher thresholds, and at thresholds down to about £14,000 per QALY.

## Supporting information

S1 Appendix Supplementary material containing data and further information on methods. (DOCX)
