# Supplementary material for: Cost-effectiveness of adjunct non-pharmacological interventions for osteoarthritis of the knee
Source: PLoS One. 2017 Mar 7;12(3):e0172749. doi: 10.1371/journal.pone.0172749 (PMC5340388; doi:10.1371/journal.pone.0172749)
Supplement: S1 Appendix — (DOCX) [file pone.0172749.s001.docx]

**Supplementary material**

Contents

[1. Study information 2](#_Toc465930585)

[2. Further detail on mapping approach 15](#_Toc465930586)

[4. Network meta-analysis methods 18](#_Toc465930587)

[4. Networks of evidence 22](#_Toc465930588)

# 1. Study information

Table S1: Study characteristics for analysis dataset

| Study 1st author, year | Intervention | N analysed | Quality of life instrument | Analysis time point (weeks) | IPD available | Adequate allocation concealment |
| --- | --- | --- | --- | --- | --- | --- |
| Adedoyin 2002 | Interferential therapy | 15 | Pain VAS | 4 | No | No |
| Adedoyin 2002 | Placebo | 15 | Pain VAS | 4 | No | No |
| Aglamis 2009 | Exercise - Aerobic | 16 | SF36 dimensions | 6 | No | No |
| Aglamis 2009 | Usual care | 9 | SF36 dimensions | 6 | No | No |
| Alcidi 2007 | Heat treatment | 20 | Pain VAS | 0.71 | No | No |
| Alcidi 2007 | TENS | 20 | Pain VAS | 0.71 | No | No |
| An 2008 | Exercise - Muscle strengthening | 11 | WOMAC | 8 | No | No |
| An 2008 | Usual care | 10 | WOMAC | 8 | No | No |
| Arazpour 2013 | Braces | 12 | Pain VAS | 6 | No | No |
| Arazpour 2013 | Insoles | 12 | Pain VAS | 6 | No | No |
| Baker 2001 | Exercise - Muscle strengthening | 19 | SF36 dimensions | 16 | No | Yes |
| Baker 2001 | Placebo | 19 | SF36 dimensions | 16 | No | Yes |
| Balint 2007 | Balneotherapy | 27 | WOMAC | 4 | No | Yes |
| Balint 2007 | Placebo | 25 | WOMAC | 4 | No | Yes |
| Bennell 2011 | Insoles | 90 | WOMAC | 52 | No | Yes |
| Bennell 2011 | Usual care | 89 | WOMAC | 52 | No | Yes |
| Berman 1999 | Acupuncture | 36 | WOMAC | 8 | No | Yes |
| Berman 1999 | Usual care | 37 | WOMAC | 8 | No | Yes |
| Berman 2004 | Acupuncture | 154 | EQ-5D | 8 | Yes | Yes |
| Berman 2004 | Sham Acupuncture | 145 | EQ-5D | 8 | Yes | Yes |
| Brismee 2007 | Usual care | 19 | WOMAC | 9 | No | No |
| Brismee 2007 | Tai Chi | 22 | WOMAC | 9 | No | No |
| Brosseau 2012 | Exercise - Aerobic | 44 | SF36 dimensions | 52.18 | No | No |
| Brosseau 2012 | Exercise - Aerobic | 44 | SF36 dimensions | 52.18 | No | No |
| Brosseau 2012 | Usual care | 41 | SF36 dimensions | 52.18 | No | No |
| Bruce-Brand 2012 | Exercise - Muscle strengthening | 10 | SF36 MCS and PCS | 8 | No | No |
| Bruce-Brand 2012 | NMES | 10 | SF36 MCS and PCS | 8 | No | No |
| Bruce-Brand 2012 | Usual care | 6 | SF36 MCS and PCS | 8 | No | No |
| Burch 2008 | Interferential therapy | 52 | WOMAC | 8 | No | Yes |
| Burch 2008 | TENS | 53 | WOMAC | 8 | No | Yes |
| Callaghan 2005 | Placebo | 9 | Pain VAS | 2 | No | No |
| Callaghan 2005 | Pulsed electrical stimulation | 9 | Pain VAS | 2 | No | No |
| Callaghan 2005 | Pulsed electrical stimulation | 9 | Pain VAS | 2 | No | No |
| Cheing 2003 | Placebo | 8 | Pain VAS | 1.43 | No | No |
| Cheing 2003 | TENS | 10 | Pain VAS | 1.43 | No | No |
| Cheing 2003 | TENS | 10 | Pain VAS | 1.43 | No | No |
| Cheing 2003 | TENS | 10 | Pain VAS | 1.43 | No | No |
| Durmus 2007 | Exercise - Muscle strengthening | 25 | Pain VAS | 4 | No | No |
| Durmus 2007 | Pulsed electrical stimulation | 25 | Pain VAS | 4 | No | No |
| Fary 2011 | Placebo | 36 | SF36 MCS and PCS | 4 | No | Yes |
| Fary 2011 | Pulsed electrical stimulation | 34 | SF36 MCS and PCS | 4 | No | Yes |
| Fischer 2005 | Placebo | 35 | Pain VAS | 6 | No | No |
| Fischer 2005 | Pulsed electromagnetic fields | 34 | Pain VAS | 6 | No | No |
| Flusser 2002 | Balneotherapy | 40 | Pain VAS | 3 | No | No |
| Flusser 2002 | Placebo | 18 | Pain VAS | 3 | No | No |
| Foroughi 2011 | Exercise - Muscle strengthening | 20 | WOMAC | 26.09 | No | No |
| Foroughi 2011 | Placebo | 25 | WOMAC | 26.09 | No | No |
| Fukuda 2011a | Placebo | 21 | Pain NRS | 3 | No | Yes |
| Fukuda 2011a | Pulsed electrical stimulation | 30 | Pain NRS | 3 | No | Yes |
| Fukuda 2011a | Pulsed electrical stimulation | 29 | Pain NRS | 3 | No | Yes |
| Fukuda 2011a | Usual care | 32 | Pain NRS | 3 | No | Yes |
| Fukuda 2011b | Laser/light therapy | 25 | Pain VAS | 6 | No | Yes |
| Fukuda 2011b | Placebo | 22 | Pain VAS | 6 | No | Yes |
| Garland 2007 | Placebo | 19 | WOMAC | 13.04 | No | Yes |
| Garland 2007 | Pulsed electrical stimulation | 39 | WOMAC | 13.04 | No | Yes |
| Grimmer 1992 | Placebo | 20 | Pain VAS | 0.14 | No | Yes |
| Grimmer 1992 | TENS | 20 | Pain VAS | 0.14 | No | Yes |
| Grimmer 1992 | TENS | 20 | Pain VAS | 0.14 | No | Yes |
| Gundog 2012 | Interferential therapy | 15 | WOMAC | 4.35 | No | No |
| Gundog 2012 | Interferential therapy | 15 | WOMAC | 4.35 | No | No |
| Gundog 2012 | Interferential therapy | 15 | WOMAC | 4.35 | No | No |
| Gundog 2012 | Placebo | 15 | WOMAC | 4.35 | No | No |
| Hasegawa 2010 | Exercise - Muscle strengthening | 14 | Pain NRS | 12 | No | No |
| Hasegawa 2010 | Usual care | 14 | Pain NRS | 12 | No | No |
| Huang 2005 | Exercise - Muscle strengthening | 25 | Pain VAS | 8 | No | Yes |
| Huang 2005 | Usual care | 28 | Pain VAS | 8 | No | Yes |
| Imoto 2012 | Exercise - Muscle strengthening | 38 | SF36 dimensions | 8 | No | Yes |
| Imoto 2012 | Usual care | 43 | SF36 dimensions | 8 | No | Yes |
| Itoh 2008a | Acupuncture | 6 | WOMAC | 5 | No | No |
| Itoh 2008a | Usual care | 6 | WOMAC | 5 | No | No |
| Itoh 2008a | TENS | 6 | WOMAC | 5 | No | No |
| Itoh 2008b | Acupuncture | 9 | WOMAC | 5 | No | No |
| Itoh 2008b | Acupuncture | 8 | WOMAC | 5 | No | No |
| Itoh 2008b | Sham Acupuncture | 7 | WOMAC | 5 | No | No |
| Jubb 2008 | Acupuncture | 31 | WOMAC | 5 | No | No |
| Jubb 2008 | Sham Acupuncture | 31 | WOMAC | 5 | No | No |
| Kang 2007 | Placebo | 28 | Pain VAS | 0.29 | No | No |
| Kang 2007 | TENS | 35 | Pain VAS | 0.29 | No | No |
| Kovar 1992 | Exercise - Aerobic | 47 | Pain VAS | 8 | No | No |
| Kovar 1992 | Usual care | 45 | Pain VAS | 8 | No | No |
| Kuptniratsaikul 2002 | Exercise - Muscle strengthening | 173 | Pain VAS | 8 | No | No |
| Kuptniratsaikul 2002 | Usual care | 193 | Pain VAS | 8 | No | No |
| Lansdown 2009 | Acupuncture | 15 | EQ-5D | 13.04 | No | Yes |
| Lansdown 2009 | Usual care | 15 | EQ-5D | 13.04 | No | Yes |
| Lee 2009 | Usual care | 15 | SF36 MCS and PCS | 8 | No | No |
| Lee 2009 | Tai Chi | 29 | SF36 MCS and PCS | 8 | No | No |
| Lewis 1994 | Placebo | 28 | Pain VAS | 3 | No | No |
| Lewis 1994 | TENS | 28 | Pain VAS | 3 | No | No |
| Lim 2010 | Exercise - Muscle strengthening | 22 | SF36 MCS and PCS | 8 | No | Yes |
| Lim 2010 | Exercise - Muscle strengthening | 24 | SF36 MCS and PCS | 8 | No | Yes |
| Lim 2010 | Usual care | 20 | SF36 MCS and PCS | 8 | No | Yes |
| Lu 2010 | Acupuncture | 10 | Pain VAS | 0.14 | No | No |
| Lu 2010 | Sham Acupuncture | 10 | Pain VAS | 0.14 | No | No |
| Lund 2008 | Exercise - Aerobic | 25 | Pain VAS | 8 | No | Yes |
| Lund 2008 | Exercise - Muscle strengthening | 27 | Pain VAS | 8 | No | Yes |
| Lund 2008 | Usual care | 27 | Pain VAS | 8 | No | Yes |
| Maillefert 2001 | Insoles | 82 | WOMAC | 4.35 | No | No |
| Maillefert 2001 | Placebo | 74 | WOMAC | 4.35 | No | No |
| Maurer 1999 | Exercise - Muscle strengthening | 49 | WOMAC | 8 | No | No |
| Maurer 1999 | Usual care | 49 | WOMAC | 8 | No | No |
| Mavrommatis 2012 | Acupuncture | 39 | SF36 MCS and PCS | 8 | No | No |
| Mavrommatis 2012 | Sham Acupuncture | 40 | SF36 MCS and PCS | 8 | No | No |
| Mavrommatis 2012 | Usual care | 38 | SF36 MCS and PCS | 8 | No | No |
| Mazzuca 2004 | Heat treatment | 26 | WOMAC | 4 | No | Yes |
| Mazzuca 2004 | Placebo | 25 | WOMAC | 4 | No | Yes |
| McCarthy 2004 | Exercise - Muscle strengthening | 80 | EQ-5D | 4 | No | Yes |
| McCarthy 2004 | Usual care | 71 | EQ-5D | 4 | No | Yes |
| Messier 2004 | Exercise - Aerobic | 69 | SF36 MCS and PCS | 39.13 | No | Yes |
| Messier 2004 | Usual care | 68 | SF36 MCS and PCS | 39.13 | No | Yes |
| Miranda-Filloy 2005 | Placebo | 10 | WOMAC | 8 | No | No |
| Miranda-Filloy 2005 | Pulsed electrical stimulation | 10 | WOMAC | 8 | No | No |
| Ng 2003 | Acupuncture | 8 | Pain NRS | 4 | No | No |
| Ng 2003 | TENS | 8 | Pain NRS | 4 | No | No |
| Nguyen 1997 | Balneotherapy | 91 | Pain VAS | 4 | No | No |
| Nguyen 1997 | Usual care | 97 | Pain VAS | 4 | No | No |
| Nigg 2006 | Insoles | 57 | WOMAC | 12 | No | No |
| Nigg 2006 | Usual care | 66 | WOMAC | 12 | No | No |
| Perlman 2006 | Manual therapy | 34 | WOMAC | 8 | No | No |
| Perlman 2006 | Usual care | 34 | WOMAC | 8 | No | No |
| Perlman 2012 | Manual therapy | 22 | WOMAC | 8 | No | Yes |
| Perlman 2012 | Manual therapy | 24 | WOMAC | 8 | No | Yes |
| Perlman 2012 | Manual therapy | 24 | WOMAC | 8 | No | Yes |
| Perlman 2012 | Manual therapy | 25 | WOMAC | 8 | No | Yes |
| Perlman 2012 | Usual care | 24 | WOMAC | 8 | No | Yes |
| Perrot 1998 | Placebo | 19 | Pain VAS | 1.29 | No | No |
| Perrot 1998 | Pulsed electromagnetic fields | 21 | Pain VAS | 1.29 | No | No |
| Pietrosimone 2009 | Ice/cooling treatment | 11 | Pain VAS | 0.14 | No | Yes |
| Pietrosimone 2009 | Usual care | 12 | Pain VAS | 0.14 | No | Yes |
| Pietrosimone 2009 | TENS | 10 | Pain VAS | 0.14 | No | Yes |
| Pipitone 2001 | Placebo | 35 | WOMAC | 6 | No | Yes |
| Pipitone 2001 | Pulsed electromagnetic fields | 34 | WOMAC | 6 | No | Yes |
| Pollard 2008 | Manual therapy | 26 | Pain VAS | 2 | No | Yes |
| Pollard 2008 | Placebo | 17 | Pain VAS | 2 | No | Yes |
| Rapp 2009 | Exercise - Muscle strengthening | 9 | Pain VAS | 8 | No | No |
| Rapp 2009 | Manual therapy | 15 | Pain VAS | 8 | No | No |
| Rapp 2009 | Usual care | 15 | Pain VAS | 8 | No | No |
| Rattanachaiyanont 2008 | Heat treatment | 50 | WOMAC | 3 | No | Yes |
| Rattanachaiyanont 2008 | Placebo | 54 | WOMAC | 3 | No | Yes |
| Salacinski 2012 | Exercise - Aerobic | 13 | WOMAC | 12 | No | Yes |
| Salacinski 2012 | Usual care | 15 | WOMAC | 12 | No | Yes |
| Salli 2010 | Exercise - Muscle strengthening | 24 | SF36 MCS and PCS | 8 | No | No |
| Salli 2010 | Exercise - Muscle strengthening | 23 | SF36 MCS and PCS | 8 | No | No |
| Salli 2010 | Usual care | 24 | SF36 MCS and PCS | 8 | No | No |
| Sangdee 2002 | Acupuncture | 46 | WOMAC | 4 | No | No |
| Sangdee 2002 | Acupuncture | 46 | WOMAC | 4 | No | No |
| Sangdee 2002 | Placebo | 45 | WOMAC | 4 | No | No |
| Sangdee 2002 | Usual care | 49 | WOMAC | 4 | No | No |
| Selfe 2008 | Placebo | 19 | WOMAC | 8 | No | Yes |
| Selfe 2008 | TENS | 18 | WOMAC | 8 | No | Yes |
| Shen 2009 | Laser/light therapy | 18 | WOMAC | 4 | No | Yes |
| Shen 2009 | Placebo | 9 | WOMAC | 4 | No | Yes |
| Sherman 2009 | Balneotherapy | 24 | WOMAC | 7 | No | No |
| Sherman 2009 | Placebo | 20 | WOMAC | 7 | No | No |
| Stelian 1992 | Laser/light therapy | 15 | Pain VAS | 1.43 | No | No |
| Stelian 1992 | Laser/light therapy | 18 | Pain VAS | 1.43 | No | No |
| Stelian 1992 | Placebo | 17 | Pain VAS | 1.43 | No | No |
| Suarez-Almazor 2010 | Acupuncture | 153 | SF12 MCS and PCS | 6 | No | Yes |
| Suarez-Almazor 2010 | Sham Acupuncture | 302 | SF12 MCS and PCS | 6 | No | Yes |
| Sukenik 1999 | Balneotherapy | 10 | Pain VAS | 2 | No | No |
| Sukenik 1999 | Balneotherapy | 10 | Pain VAS | 2 | No | No |
| Sukenik 1999 | Balneotherapy | 7 | Pain VAS | 2 | No | No |
| Sukenik 1999 | Placebo | 9 | Pain VAS | 2 | No | No |
| Tascioglu 2004 | Laser/light therapy | 20 | WOMAC | 3 | No | No |
| Tascioglu 2004 | Laser/light therapy | 20 | WOMAC | 3 | No | No |
| Tascioglu 2004 | Placebo | 20 | WOMAC | 3 | No | No |
| Thamsborg 2005 | Placebo | 41 | WOMAC | 6 | No | No |
| Thamsborg 2005 | Pulsed electromagnetic fields | 42 | WOMAC | 6 | No | No |
| Thorstensson 2005 | Exercise - Aerobic | 28 | SF36 MCS and PCS | 6 | No | No |
| Thorstensson 2005 | Usual care | 28 | SF36 MCS and PCS | 6 | No | No |
| Tishler 2004 | Balneotherapy | 44 | Pain VAS | 6 | No | No |
| Tishler 2004 | Usual care | 24 | Pain VAS | 6 | No | No |
| Toda 2008 | Insoles | 43 | Pain VAS | 12 | No | Yes |
| Toda 2008 | Insoles | 41 | Pain VAS | 12 | No | Yes |
| Toda 2008 | Placebo | 38 | Pain VAS | 12 | No | Yes |
| Topp 2002 | Exercise - Muscle strengthening | 35 | WOMAC | 16 | No | No |
| Topp 2002 | Exercise - Muscle strengthening | 32 | WOMAC | 16 | No | No |
| Topp 2002 | Usual care | 35 | WOMAC | 16 | No | No |
| Trock 1994 | Placebo | 44 | Pain VAS | 5 | No | Yes |
| Trock 1994 | Pulsed electromagnetic fields | 40 | Pain VAS | 5 | No | Yes |
| Tucker 2003 | Manual therapy | 30 | Pain VAS | 3 | No | No |
| Tucker 2003 | Usual care | 30 | Pain VAS | 3 | No | No |
| Tukmachi 2004 | Acupuncture | 9 | Pain VAS | 5 | No | Yes |
| Tukmachi 2004 | Acupuncture | 10 | Pain VAS | 5 | No | Yes |
| Tukmachi 2004 | Usual care | 10 | Pain VAS | 5 | No | Yes |
| Vance 2012 | Placebo | 25 | Pain VAS | 0.14 | No | Yes |
| Vance 2012 | TENS | 25 | Pain VAS | 0.14 | No | Yes |
| Vance 2012 | TENS | 25 | Pain VAS | 0.14 | No | Yes |
| Vas 2004 | Acupuncture | 47 | WOMAC | 12 | Yes | Yes |
| Vas 2004 | Sham Acupuncture | 43 | WOMAC | 12 | Yes | Yes |
| Wang 2009 | Usual care | 20 | SF36 MCS and PCS | 12 | No | Yes |
| Wang 2009 | Tai Chi | 20 | SF36 MCS and PCS | 12 | No | Yes |
| Wigler 1995 | Balneotherapy | 11 | Pain VAS | 4 | No | No |
| Wigler 1995 | Balneotherapy | 10 | Pain VAS | 4 | No | No |
| Wigler 1995 | Placebo | 12 | Pain VAS | 4 | No | No |
| Williamson 2007 | Acupuncture | 59 | WOMAC | 8 | Yes | Yes |
| Williamson 2007 | Exercise - Muscle strengthening | 53 | WOMAC | 8 | Yes | Yes |
| Williamson 2007 | Usual care | 49 | WOMAC | 8 | Yes | Yes |
| Witt 2005 | Acupuncture | 145 | SF36 dimensions | 8 | Yes | Yes |
| Witt 2005 | Sham Acupuncture | 73 | SF36 dimensions | 8 | Yes | Yes |
| Witt 2005 | Usual care | 67 | SF36 dimensions | 8 | Yes | Yes |
| Witt 2006 | Acupuncture | 250 | SF36 dimensions | 12 | Yes | Yes |
| Witt 2006 | Usual care | 244 | SF36 dimensions | 12 | Yes | Yes |
| Wolsko 2004 | Placebo | 13 | WOMAC | 6 | No | Yes |
| Wolsko 2004 | Static magnets | 13 | WOMAC | 6 | No | Yes |
| Yip 2007 | Usual care | 70 | Pain VAS | 1 | No | No |
| Yip 2007 | Tai Chi | 79 | Pain VAS | 1 | No | No |
| Yurtkuran 2006 | Balneotherapy | 27 | WOMAC | 2 | No | Yes |
| Yurtkuran 2006 | Placebo | 28 | WOMAC | 2 | No | Yes |
| Zizic 1995 | Placebo | 33 | Pain VAS | 4 | No | No |
| Zizic 1995 | Pulsed electrical stimulation | 38 | Pain VAS | 4 | No | No |

Table S2: Data used in network meta-analysis

| **Study 1st author, year** | **Intervention** | **Quality of life instrument** | **Baseline**  **EQ-5D, mean(se)** | **Follow-up EQ-5D, mean(se)** |
| --- | --- | --- | --- | --- |
| Adedoyin 2002 | Interferential therapy | Pain VAS | 0.48 ( NA) | 0.69 ( NA) |
| Adedoyin 2002 | Placebo | Pain VAS | 0.49 ( NA) | 0.63 ( NA) |
| Aglamis 2009 | Usual care | SF36 dimensions | 0.41 (0.08) | 0.50 (0.08) |
| Aglamis 2009 | Exercise - Aerobic | SF36 dimensions | 0.68 (0.05) | 0.87 (0.03) |
| Alcidi 2007 | Heat treatment | Pain VAS | 0.53 (0.04) | 0.59 (0.06) |
| Alcidi 2007 | TENS | Pain VAS | 0.56 (0.05) | 0.60 (0.05) |
| An 2008 | Usual care | WOMAC | 0.70 (0.03) | 0.62 (0.08) |
| An 2008 | Exercise - Muscle strengthening | WOMAC | 0.66 (0.06) | 0.68 (0.06) |
| Arazpour 2013 | Insoles | Pain VAS | 0.52 (0.02) | 0.61 (0.02) |
| Arazpour 2013 | Braces | Pain VAS | 0.54 (0.02) | 0.61 (0.02) |
| Baker 2001 | Placebo | SF36 dimensions | 0.76 (0.04) | 0.75 (0.05) |
| Baker 2001 | Exercise - Muscle strengthening | SF36 dimensions | 0.69 (0.05) | 0.78 (0.04) |
| Balint 2007 | Balneotherapy | WOMAC | 0.56 (0.01) | 0.60 (0.01) |
| Balint 2007 | Placebo | WOMAC | 0.56 (0.01) | 0.65 (0.01) |
| Bennell 2011 | Usual care | WOMAC | 0.61 (0.02) | 0.64 (0.02) |
| Bennell 2011 | Insoles | WOMAC | 0.60 (0.02) | 0.63 (0.02) |
| Berman 1999 | Acupuncture | WOMAC | 0.47 (0.05) | 0.64 (0.03) |
| Berman 1999 | Usual care | WOMAC | 0.46 (0.04) | 0.46 (0.05) |
| Berman 2004 | Acupuncture | EQ-5D | 0.61 (0.02) | 0.68 (0.01) |
| Berman 2004 | Sham Acupuncture | EQ-5D | 0.62 (0.02) | 0.69 (0.01) |
| Brismee 2007 | Tai Chi | WOMAC | 0.34 (0.08) | 0.48 (0.08) |
| Brismee 2007 | Usual care | WOMAC | 0.41 (0.07) | 0.38 (0.07) |
| Brosseau 2012 | Usual care | SF36 dimensions | 0.81 (0.02) | 0.82 (0.02) |
| Brosseau 2012 | Exercise - Aerobic | SF36 dimensions | 0.82 (0.02) | 0.81 (0.02) |
| Brosseau 2012 | Exercise - Aerobic | SF36 dimensions | 0.79 (0.02) | 0.81 (0.02) |
| Bruce-Brand 2012 | Usual care | SF36 MCS and PCS | NA ( NA) | 0.42 (0.26) |
| Bruce-Brand 2012 | Exercise - Muscle strengthening | SF36 MCS and PCS | NA ( NA) | 0.46 (0.17) |
| Bruce-Brand 2012 | NMES | SF36 MCS and PCS | NA ( NA) | 0.44 (0.15) |
| Burch 2008 | TENS | WOMAC | 0.48 (0.04) | 0.57 (0.04) |
| Burch 2008 | Interferential therapy | WOMAC | 0.49 (0.04) | 0.64 (0.02) |
| Callaghan 2005 | Pulsed electrical stimulation | Pain VAS | 0.54 (0.08) | 0.56 (0.09) |
| Callaghan 2005 | Pulsed electrical stimulation | Pain VAS | 0.57 (0.11) | 0.58 (0.12) |
| Callaghan 2005 | Placebo | Pain VAS | 0.55 (0.02) | 0.54 (0.07) |
| Cheing 2003 | TENS | Pain VAS | 0.59 (0.04) | 0.67 (0.04) |
| Cheing 2003 | TENS | Pain VAS | 0.57 (0.03) | 0.71 (0.02) |
| Cheing 2003 | TENS | Pain VAS | 0.56 (0.04) | 0.68 (0.03) |
| Cheing 2003 | Placebo | Pain VAS | 0.58 (0.03) | 0.59 (0.03) |
| Durmus 2007 | Pulsed electrical stimulation | Pain VAS | 0.57 ( NA) | 0.68 ( NA) |
| Durmus 2007 | Exercise - Muscle strengthening | Pain VAS | 0.55 ( NA) | 0.67 ( NA) |
| Fary 2011 | Placebo | SF36 MCS and PCS | 0.58 (0.04) | 0.64 ( NA) |
| Fary 2011 | Pulsed electrical stimulation | SF36 MCS and PCS | 0.59 (0.04) | 0.66 ( NA) |
| Fischer 2005 | Pulsed electromagnetic fields | Pain VAS | 0.56 (0.02) | 0.58 (0.03) |
| Fischer 2005 | Placebo | Pain VAS | 0.55 (0.02) | 0.62 (0.03) |
| Flusser 2002 | Balneotherapy | Pain VAS | 0.53 (0.03) | 0.55 (0.03) |
| Flusser 2002 | Placebo | Pain VAS | 0.54 (0.05) | 0.53 (0.05) |
| Foroughi 2011 | Exercise - Muscle strengthening | WOMAC | 0.65 (0.04) | 0.70 (0.02) |
| Foroughi 2011 | Placebo | WOMAC | 0.62 (0.04) | 0.66 (0.03) |
| Fukuda 2011a | Usual care | Pain NRS | 0.58 (0.04) | 0.60 (0.04) |
| Fukuda 2011a | Placebo | Pain NRS | 0.49 (0.04) | 0.54 (0.05) |
| Fukuda 2011a | Pulsed electrical stimulation | Pain NRS | 0.53 (0.05) | 0.68 (0.03) |
| Fukuda 2011a | Pulsed electrical stimulation | Pain NRS | 0.55 (0.05) | 0.64 (0.04) |
| Fukuda 2011b | Placebo | Pain VAS | 0.54 (0.02) | 0.57 (0.04) |
| Fukuda 2011b | Laser/light therapy | Pain VAS | 0.54 (0.04) | 0.59 (0.02) |
| Garland 2007 | Pulsed electrical stimulation | WOMAC | 0.46 (0.05) | 0.54 (0.06) |
| Garland 2007 | Placebo | WOMAC | 0.53 (0.05) | 0.52 (0.07) |
| Grimmer 1992 | TENS | Pain VAS | 0.52 (0.06) | 0.65 (0.05) |
| Grimmer 1992 | TENS | Pain VAS | 0.55 (0.05) | 0.68 (0.03) |
| Grimmer 1992 | Placebo | Pain VAS | 0.54 (0.05) | 0.62 (0.06) |
| Gundog 2012 | Interferential therapy | WOMAC | 0.31 (0.06) | 0.73 (0.01) |
| Gundog 2012 | Interferential therapy | WOMAC | 0.24 (0.08) | 0.74 (0.01) |
| Gundog 2012 | Interferential therapy | WOMAC | 0.22 (0.06) | 0.71 (0.03) |
| Gundog 2012 | Placebo | WOMAC | 0.27 (0.06) | 0.41 (0.05) |
| Hasegawa 2010 | Exercise - Muscle strengthening | Pain NRS | 0.64 (0.05) | 0.72 (0.05) |
| Hasegawa 2010 | Usual care | Pain NRS | 0.68 (0.05) | 0.67 (0.06) |
| Huang 2005 | Exercise - Muscle strengthening | Pain VAS | 0.58 (0.03) | 0.61 (0.01) |
| Huang 2005 | Usual care | Pain VAS | 0.58 (0.03) | 0.60 (0.03) |
| Imoto 2012 | Usual care | SF36 dimensions | 0.52 (0.04) | 0.60 (0.04) |
| Imoto 2012 | Exercise - Muscle strengthening | SF36 dimensions | 0.52 (0.03) | 0.66 (0.04) |
| Itoh 2008a | Acupuncture | WOMAC | 0.45 (0.08) | 0.59 (0.05) |
| Itoh 2008a | TENS | WOMAC | 0.44 (0.13) | 0.60 (0.04) |
| Itoh 2008a | Usual care | WOMAC | 0.49 (0.06) | 0.52 (0.06) |
| Itoh 2008b | Acupuncture | WOMAC | 0.39 (0.08) | 0.54 (0.03) |
| Itoh 2008b | Acupuncture | WOMAC | 0.46 (0.08) | 0.63 (0.05) |
| Itoh 2008b | Sham Acupuncture | WOMAC | 0.45 (0.06) | 0.48 (0.05) |
| Jubb 2008 | Sham Acupuncture | WOMAC | 0.41 (0.07) | 0.48 (0.06) |
| Jubb 2008 | Acupuncture | WOMAC | 0.37 (0.06) | 0.54 (0.05) |
| Kang 2007 | TENS | Pain VAS | 0.56 ( NA) | 0.59 ( NA) |
| Kang 2007 | Placebo | Pain VAS | 0.55 ( NA) | 0.57 ( NA) |
| Kovar 1992 | Exercise - Aerobic | Pain VAS | 0.57 (0.03) | 0.61 (0.02) |
| Kovar 1992 | Usual care | Pain VAS | 0.58 (0.03) | 0.58 (0.03) |
| Kuptniratsaikul 2002 | Exercise - Muscle strengthening | Pain VAS | 0.56 (0.01) | 0.60 (0.02) |
| Kuptniratsaikul 2002 | Usual care | Pain VAS | 0.55 (0.01) | 0.57 (0.02) |
| Lansdown 2009 | Usual care | EQ-5D | 0.67 (0.04) | 0.66 (0.06) |
| Lansdown 2009 | Acupuncture | EQ-5D | 0.61 (0.06) | 0.71 (0.07) |
| Lee 2009 | Usual care | SF36 MCS and PCS | 0.58 (0.10) | 0.56 (0.12) |
| Lee 2009 | Tai Chi | SF36 MCS and PCS | 0.51 (0.09) | 0.49 (0.09) |
| Lewis 1994 | TENS | Pain VAS | NA ( NA) | 0.58 ( NA) |
| Lewis 1994 | Placebo | Pain VAS | NA ( NA) | 0.56 ( NA) |
| Lim 2010 | Usual care | SF36 MCS and PCS | 0.51 (0.07) | 0.54 (0.08) |
| Lim 2010 | Exercise - Muscle strengthening | SF36 MCS and PCS | 0.57 (0.04) | 0.65 (0.03) |
| Lim 2010 | Exercise - Muscle strengthening | SF36 MCS and PCS | 0.53 (0.06) | 0.63 (0.03) |
| Lu 2010 | Acupuncture | Pain VAS | 0.56 (0.03) | 0.60 (0.03) |
| Lu 2010 | Sham Acupuncture | Pain VAS | 0.57 (0.04) | 0.58 (0.04) |
| Lund 2008 | Usual care | Pain VAS | 0.58 (0.05) | 0.55 (0.04) |
| Lund 2008 | Exercise - Muscle strengthening | Pain VAS | 0.54 (0.03) | 0.56 (0.04) |
| Lund 2008 | Exercise - Aerobic | Pain VAS | 0.57 (0.06) | 0.57 (0.04) |
| Maillefert 2001 | Insoles | WOMAC | 0.48 (0.04) | 0.45 (0.04) |
| Maillefert 2001 | Placebo | WOMAC | 0.47 (0.04) | 0.49 (0.04) |
| Maurer 1999 | Usual care | WOMAC | 0.60 ( NA) | 0.64 ( NA) |
| Maurer 1999 | Exercise - Muscle strengthening | WOMAC | 0.62 ( NA) | 0.67 ( NA) |
| Mavrommatis 2012 | Usual care | SF36 MCS and PCS | 0.46 (0.04) | 0.60 (0.02) |
| Mavrommatis 2012 | Sham Acupuncture | SF36 MCS and PCS | 0.43 (0.05) | 0.61 (0.02) |
| Mavrommatis 2012 | Acupuncture | SF36 MCS and PCS | 0.48 (0.03) | 0.72 (0.02) |
| Mazzuca 2004 | Heat treatment | WOMAC | 0.46 (0.07) | 0.52 (0.06) |
| Mazzuca 2004 | Placebo | WOMAC | 0.47 (0.07) | 0.53 (0.05) |
| McCarthy 2004 | Usual care | EQ-5D | 0.50 ( NA) | 0.52 ( NA) |
| McCarthy 2004 | Exercise - Muscle strengthening | EQ-5D | 0.54 ( NA) | 0.60 ( NA) |
| Messier 2004 | Usual care | SF36 MCS and PCS | 0.55 (0.03) | 0.61 (0.02) |
| Messier 2004 | Exercise - Aerobic | SF36 MCS and PCS | 0.58 (0.02) | 0.63 (0.02) |
| Miranda-Filloy 2005 | Pulsed electrical stimulation | WOMAC | 0.51 ( NA) | 0.69 ( NA) |
| Miranda-Filloy 2005 | Placebo | WOMAC | 0.46 ( NA) | 0.55 ( NA) |
| Ng 2003 | Acupuncture | Pain NRS | 0.65 (0.05) | 0.70 (0.04) |
| Ng 2003 | TENS | Pain NRS | 0.67 (0.04) | 0.69 (0.04) |
| Nguyen 1997 | Usual care | Pain VAS | 0.58 (0.02) | 0.58 (0.02) |
| Nguyen 1997 | Balneotherapy | Pain VAS | 0.57 (0.02) | 0.62 (0.02) |
| Nigg 2006 | Insoles | WOMAC | 0.61 (0.03) | 0.66 (0.02) |
| Nigg 2006 | Usual care | WOMAC | 0.60 (0.03) | 0.65 (0.03) |
| Perlman 2006 | Manual therapy | WOMAC | 0.49 (0.06) | 0.65 (0.04) |
| Perlman 2006 | Usual care | WOMAC | 0.42 (0.06) | 0.47 (0.06) |
| Perlman 2012 | Manual therapy | WOMAC | 0.44 (0.08) | 0.60 (0.05) |
| Perlman 2012 | Manual therapy | WOMAC | 0.47 (0.07) | 0.62 (0.05) |
| Perlman 2012 | Manual therapy | WOMAC | 0.44 (0.07) | 0.64 (0.04) |
| Perlman 2012 | Manual therapy | WOMAC | 0.49 (0.07) | 0.66 (0.04) |
| Perlman 2012 | Usual care | WOMAC | 0.45 (0.06) | 0.52 (0.05) |
| Perrot 1998 | Pulsed electromagnetic fields | Pain VAS | 0.52 ( NA) | 0.60 ( NA) |
| Perrot 1998 | Placebo | Pain VAS | 0.50 ( NA) | 0.54 ( NA) |
| Pietrosimone 2009 | TENS | Pain VAS | 0.68 (0.04) | 0.70 (0.02) |
| Pietrosimone 2009 | Ice/cooling treatment | Pain VAS | 0.67 (0.04) | 0.69 (0.04) |
| Pietrosimone 2009 | Usual care | Pain VAS | 0.67 (0.04) | 0.67 (0.05) |
| Pipitone 2001 | Placebo | WOMAC | 0.44 (0.05) | 0.46 (0.05) |
| Pipitone 2001 | Pulsed electromagnetic fields | WOMAC | 0.44 (0.07) | 0.49 (0.06) |
| Pollard 2008 | Manual therapy | Pain VAS | 0.63 (0.04) | 0.67 (0.03) |
| Pollard 2008 | Placebo | Pain VAS | 0.62 (0.06) | 0.63 (0.05) |
| Rapp 2009 | Exercise - Muscle strengthening | Pain VAS | NA ( NA) | 0.67 (0.05) |
| Rapp 2009 | Manual therapy | Pain VAS | NA ( NA) | 0.66 (0.04) |
| Rapp 2009 | Usual care | Pain VAS | NA ( NA) | 0.64 (0.05) |
| Rattanachaiyanont 2008 | Heat treatment | WOMAC | 0.60 (0.03) | 0.66 (0.02) |
| Rattanachaiyanont 2008 | Placebo | WOMAC | 0.58 (0.03) | 0.66 (0.02) |
| Salacinski 2012 | Usual care | WOMAC | 0.66 (0.04) | 0.64 (0.05) |
| Salacinski 2012 | Exercise - Aerobic | WOMAC | 0.65 (0.04) | 0.71 (0.02) |
| Salli 2010 | Usual care | SF36 MCS and PCS | 0.54 (0.07) | 0.59 (0.05) |
| Salli 2010 | Exercise - Muscle strengthening | SF36 MCS and PCS | 0.55 (0.08) | 0.62 (0.05) |
| Salli 2010 | Exercise - Muscle strengthening | SF36 MCS and PCS | 0.53 (0.09) | 0.60 (0.08) |
| Sangdee 2002 | Acupuncture | WOMAC | 0.42 (0.06) | 0.65 (0.03) |
| Sangdee 2002 | Acupuncture | WOMAC | 0.42 (0.06) | 0.65 (0.03) |
| Sangdee 2002 | Usual care | WOMAC | 0.44 (0.05) | 0.63 (0.03) |
| Sangdee 2002 | Placebo | WOMAC | 0.44 (0.05) | 0.60 (0.03) |
| Selfe 2008 | Placebo | WOMAC | 0.45 (0.08) | 0.63 (0.05) |
| Selfe 2008 | TENS | WOMAC | 0.44 (0.10) | 0.64 (0.05) |
| Shen 2009 | Laser/light therapy | WOMAC | 0.58 (0.06) | 0.69 (0.03) |
| Shen 2009 | Placebo | WOMAC | 0.66 (0.03) | 0.73 (0.01) |
| Sherman 2009 | Placebo | WOMAC | 0.40 (0.09) | 0.50 (0.08) |
| Sherman 2009 | Balneotherapy | WOMAC | 0.36 (0.08) | 0.49 (0.08) |
| Stelian 1992 | Laser/light therapy | Pain VAS | 0.53 (0.06) | 0.62 (0.06) |
| Stelian 1992 | Laser/light therapy | Pain VAS | 0.51 (0.05) | 0.63 (0.05) |
| Stelian 1992 | Placebo | Pain VAS | 0.54 (0.05) | 0.54 (0.05) |
| Suarez-Almazor 2010 | Sham Acupuncture | SF12 MCS and PCS | 0.61 (0.02) | 0.69 (0.02) |
| Suarez-Almazor 2010 | Acupuncture | SF12 MCS and PCS | 0.61 (0.02) | 0.71 (0.02) |
| Sukenik 1999 | Balneotherapy | Pain VAS | 0.54 (0.05) | 0.55 (0.04) |
| Sukenik 1999 | Balneotherapy | Pain VAS | 0.53 (0.03) | 0.56 (0.05) |
| Sukenik 1999 | Balneotherapy | Pain VAS | 0.52 (0.03) | 0.61 (0.06) |
| Sukenik 1999 | Placebo | Pain VAS | 0.54 (0.05) | 0.54 (0.04) |
| Tascioglu 2004 | Laser/light therapy | WOMAC | 0.46 (0.05) | 0.48 (0.05) |
| Tascioglu 2004 | Laser/light therapy | WOMAC | 0.42 (0.05) | 0.45 (0.05) |
| Tascioglu 2004 | Placebo | WOMAC | 0.43 (0.06) | 0.46 (0.05) |
| Thamsborg 2005 | Pulsed electromagnetic fields | WOMAC | 0.46 (0.04) | 0.54 (0.03) |
| Thamsborg 2005 | Placebo | WOMAC | 0.42 (0.05) | 0.48 (0.05) |
| Thorstensson 2005 | Usual care | SF36 MCS and PCS | 0.72 ( NA) | 0.73 ( NA) |
| Thorstensson 2005 | Exercise - Aerobic | SF36 MCS and PCS | 0.71 ( NA) | 0.74 ( NA) |
| Tishler 2004 | Balneotherapy | Pain VAS | 0.53 (0.03) | 0.66 (0.03) |
| Tishler 2004 | Usual care | Pain VAS | 0.52 (0.04) | 0.53 (0.04) |
| Toda 2008 | Placebo | Pain VAS | 0.59 (0.03) | 0.58 (0.02) |
| Toda 2008 | Insoles | Pain VAS | 0.61 (0.03) | 0.60 (0.03) |
| Toda 2008 | Insoles | Pain VAS | 0.59 (0.03) | 0.60 (0.04) |
| Topp 2002 | Exercise - Muscle strengthening | WOMAC | 0.35 (0.06) | 0.40 (0.09) |
| Topp 2002 | Exercise - Muscle strengthening | WOMAC | 0.40 (0.06) | 0.45 (0.05) |
| Topp 2002 | Usual care | WOMAC | 0.41 (0.06) | 0.39 (0.06) |
| Trock 1994 | Placebo | Pain VAS | 0.53 (0.03) | 0.57 (0.03) |
| Trock 1994 | Pulsed electromagnetic fields | Pain VAS | 0.51 (0.03) | 0.59 (0.03) |
| Tucker 2003 | Manual therapy | Pain VAS | 0.62 (0.03) | 0.69 (0.02) |
| Tucker 2003 | Usual care | Pain VAS | 0.63 (0.04) | 0.69 (0.02) |
| Tukmachi 2004 | Acupuncture | Pain VAS | 0.54 (0.06) | 0.63 (0.08) |
| Tukmachi 2004 | Acupuncture | Pain VAS | 0.54 (0.05) | 0.67 (0.06) |
| Tukmachi 2004 | Usual care | Pain VAS | 0.51 (0.04) | 0.52 (0.06) |
| Vance 2012 | TENS | Pain VAS | 0.65 (0.04) | 0.68 (0.04) |
| Vance 2012 | TENS | Pain VAS | 0.64 (0.04) | 0.66 (0.04) |
| Vance 2012 | Placebo | Pain VAS | 0.64 (0.04) | 0.68 (0.03) |
| Vas 2004 | Acupuncture | WOMAC | 0.40 (0.06) | 0.75 (0.04) |
| Vas 2004 | Sham Acupuncture | WOMAC | 0.35 (0.06) | 0.56 (0.06) |
| Wang 2009 | Usual care | SF36 MCS and PCS | 0.50 (0.06) | 0.56 (0.05) |
| Wang 2009 | Tai Chi | SF36 MCS and PCS | 0.58 (0.05) | 0.71 (0.04) |
| Wigler 1995 | Balneotherapy | Pain VAS | 0.51 ( NA) | 0.55 ( NA) |
| Wigler 1995 | Balneotherapy | Pain VAS | 0.51 ( NA) | 0.56 ( NA) |
| Wigler 1995 | Placebo | Pain VAS | 0.51 ( NA) | 0.54 ( NA) |
| Williamson 2007 | Acupuncture | WOMAC | 0.44 (0.04) | 0.43 (0.04) |
| Williamson 2007 | Exercise - Muscle strengthening | WOMAC | 0.43 (0.05) | 0.46 (0.05) |
| Williamson 2007 | Usual care | WOMAC | 0.43 (0.04) | 0.36 (0.04) |
| Witt 2005 | Acupuncture | SF36 dimensions | 0.57 (0.02) | 0.70 (0.02) |
| Witt 2005 | Sham Acupuncture | SF36 dimensions | 0.55 (0.02) | 0.62 (0.03) |
| Witt 2005 | Usual care | SF36 dimensions | 0.56 (0.03) | 0.57 (0.02) |
| Witt 2006 | Acupuncture | SF36 dimensions | 0.57 (0.01) | 0.67 (0.02) |
| Witt 2006 | Usual care | SF36 dimensions | 0.57 (0.01) | 0.56 (0.02) |
| Wolsko 2004 | Static magnets | WOMAC | 0.47 (0.12) | 0.64 (0.07) |
| Wolsko 2004 | Placebo | WOMAC | 0.48 ( NA) | 0.66 ( NA) |
| Yip 2007 | Usual care | Pain VAS | 0.59 (0.02) | 0.59 (0.03) |
| Yip 2007 | Tai Chi | Pain VAS | 0.57 (0.02) | 0.61 (0.02) |
| Yurtkuran 2006 | Placebo | WOMAC | 0.44 (0.04) | 0.58 (0.04) |
| Yurtkuran 2006 | Balneotherapy | WOMAC | 0.47 (0.06) | 0.64 (0.03) |
| Zizic 1995 | Pulsed electrical stimulation | Pain VAS | 0.53 ( NA) | 0.59 ( NA) |
| Zizic 1995 | Placebo | Pain VAS | 0.54 ( NA) | 0.58 ( NA) |

# 2. Further detail on mapping approach

In a number of studies, data were reported for multiple health related quality of life instruments for which mappings were available. The preferred instrument was selected from these studies based on the extent to which the instrument was expected to reflect all dimensions of the EQ-5D. This resulted in the following hierarchy: EQ-5D preference values; SF-36 dimension scores; SF36 mental and physical component summary scores (SF36 MCS and PCS); SF-12 mental and physical component summary scores (SF12 MCS and PCS); WOMAC total score; visual analogue scale (pain VAS) measures of pain; and numerical rating scale (pain NRS) measure of pain.

The following published mapping algorithms were applied to data collected using these instruments to derive EQ-5D estimates.

#### SF-36 to EQ-5D

Those studies that used the SF-36 questionnaires, reported its results either as scores for the 8 dimensions of the instrument or for the physical and mental component summary scores. The mapping algorithm published by Rowen *et al* [1] was applied for those studies that reported results for the 8 dimensions, and the mapping algorithm published by Maund *et al* [2] for those studies that reported the physical and mental component summary scores. Rowen *et al* mapping coefficients were obtained from a generalised least squares regression of individual patient-level EQ‑5D scores against the values of the 8 dimensions, their squares and their interactions as this was identified as the preferred model by the authors. The Rowen *et al* study analysed data from a wide range of inpatients and outpatients at Cardiff and Vale NHS Hospitals Trust. Maund *et al* mapping coefficients were obtained from an ordinary least squares (OLS) regression of individual patient level EQ-5D scores against the values of the physical and mental component summary scores[2] , using data from patients with rotator cuff disease in primary care recruited in the SAPPHIRE trial. The authors estimated five models: three OLS regressions (one with main effects for physical and mental component summary scores only, another adding squared terms, and a third adding both squared and interaction terms), one Tobit regression and one censored least absolute deviations (CLAD) model (both the Tobit and CLAD models included main effects, squared terms and interaction terms). All models performed similarly (with mean absolute errors of 0.18-0.19). Given this, for simplicity OLS models were preferred and the model including the main effects, their squares and their interaction was used on the basis of marginal improvements to explanatory power and model fit. The analysis of 1, 3 and 12 month data was used as this included more data.

#### SF-12 to EQ-5D

Those studies that used the SF-12 questionnaire, reported its results in terms of physical and mental component summary scores. We used the mapping algorithm published by Gray *et al* [3], which was obtained from a multinomial logistic regression model of individual patient-level EQ‑5D scores against the values of the two summary scores, their squares and an interaction term. This model is based on an analysis of data from the Medical Expenditure Panel Survey of non-institutionalised US civilians.

#### WOMAC to EQ-5D

Barton *et al* [4] developed a series of algorithms to map the WOMAC instrument on to EQ-5D scores. Using data from patients that participated in a randomised controlled trial of lifestyle interventions for knee pain, the authors conducted a series of OLS regressions, relating the EQ-5D index value to various possible ways in which the WOMAC questionnaire may be reported. Five models were estimated in total: the first had total WOMAC score as the only explanatory variable; the second used the WOMAC pain, stiffness and functioning subscales; the third used total WOMAC and total WOMAC squared; the fourth included pain, stiffness and functioning, their interactions and their squares; while the final model was the best fitting of the previous four plus age and gender. The preferred model reported by Barton *et al* included total WOMAC, total WOMAC squared, age, age squared and gender. This model was therefore applied to the individual patient data. Due to variable reporting of age and gender across studies, for aggregate data studies the model including total WOMAC and total WOMAC squared was used.

#### Pain to EQ-5D

Mapping algorithms were available to map pain measured using either a numerical rating scale (NRS) or a visual analogue score (VAS). The HTA report by Maund *et al [2]* that provided the mapping algorithm for the SF-36 mental and physical composite summary scores, also provided an algorithm to map pain VAS to EQ-5D scores. The authors estimated four models: two OLS regressions (one with pain VAS, and another with pain VAS and its squared term), one Tobit regression and one censored least absolute deviations (CLAD) model, using pain VAS and its squared term as explanatory variables. All models performed similarly (with mean absolute errors of 0.18-0.20). Given this, for simplicity OLS models were preferred and the model including the main effects and their squares was used. Although there were no observed differences in model fit or explanatory power between the model with and without the squared term, it seemed plausible that the relationship between pain VAS and EQ-5D scores was non-linear. The analysis of 1, 3 and 12 month data was used as this included more data.

A mapping algorithm from the 11-point Pain Intensity Numerical Rating Scale (PI-NRS-11), ranging from 0 (‘no pain’) to 10 (‘pain as bad as you can imagine’), to the EQ-5D was available from Gu *et al* [5]. The authors used survey data from a US sample of patients with neuropathic pain. The authors estimated two mapping algorithms. The first related the EQ-5D index score against a set of pain NRS dummy variables using OLS, and the second used an ordered logistic regression model to predict the response levels (i.e. 1,2,3) for each of the EQ-5D dimensions using the same explanatory variables. Models were run with and without patient age, gender and pain duration as independent variables. The OLS model was used in the current analysis as it had a better fit compared to the ordered logistic model. The reduced model which excluded age, gender and disease duration was used given variable reporting of this information across studies.

*Mapping using aggregate data*

Statistical mapping is typically conducted at the individual patient level. When only published articles were available, health-related quality of life dimension score data were simulated using a multivariate normal distribution (parameterised using the published mean and variance, and correlations obtained from the individual patient data studies). Samples of health related quality of life measures and the associated mapped EQ-5D values were truncated to the minimum or maximum possible values. Uncertainty introduced by the limited explanatory power of the mapping algorithms was reflected in the EQ-5D variance using the R^2^ adjusted estimator [6].

# 4. Network meta-analysis methods

Bayesian methods were used in this analysis as they offer a high degree of flexibility with respect to model structure.

Individual patient data were included in the model using an analysis of covariance (ANCOVA) network meta-analysis model:

| (E1) |
| --- |
|  |

$$Y_{\mathrm{ikjt}}\sim N(\theta_{ikj,}\sigma_{j}^{2})$$

$$\theta_{\mathrm{ikj}}=\left\{ \begin{aligned} \alpha_{\mathrm{bj}}+\beta_{j0}Y_{ikj0} if k=b \\ \alpha_{\mathrm{bj}}+\beta_{j0}Y_{ikj0}+\delta_{\mathrm{kbj}} if k>b \end{aligned} \right.$$

where $Y_{\mathrm{ijkt}}$ and $Y_{ijk0}$ are the values of the (continuous) outcome at time point *t* and baseline ($t=0$), respectively, for participant *i* in treatment arm *k* of study *j*. $\sigma_{j}^{2}$ represents the study-level variance. The quantity $\alpha_{\mathrm{bj}}$ represents the outcome for the baseline treatment *k=b* in study *j* for a patient with a baseline EQ-5D score $(Y_{ikj0})$of zero (this is study specific to respect within-study randomisation). The parameter $\beta_{j0}$ represents a study-specific estimate of the impact of baseline EQ-5D on final outcome. Each $\delta_{\mathrm{kbj}}$represents the treatment effect for treatment *k* relative to treatment *b* in trial *j*.

The aggregate data are included in a model which adjusts for the potential bias caused by differences in outcomes between treatments at baseline or “baseline imbalance” (where these differences are denoted as $Y_{kbj0}(=Y_{kj0}-Y_{bj0})$):

$$Y_{\mathrm{kjt}}\sim N(\theta_{kj,}\sigma_{\mathrm{kj}}^{2})$$

| (E2) |
| --- |

$$\theta_{\mathrm{jk}}=\left\{ \begin{aligned} \alpha_{\mathrm{bj}} if k=b \\ \alpha_{\mathrm{bj}}+\beta_{\mathrm{pred}}Y_{kbj0}+\delta_{\mathrm{kbj}} if k>b \end{aligned} \right.$$

where $Y_{\mathrm{kjt}}$ and $\sigma_{\mathrm{kj}}^{2}$ are the mean and variance for the outcome for treatment arm *k* of study *j* at time *t*.

This model for the aggregate data adjusts the treatment effects to emulate the ANCOVA model results. This is achieved by considering the reported unadjusted results as being subject to omitted variable bias, where the omitted variable is baseline EQ-5D. The magnitude of omitted variable bias can be estimated as the product of the coefficient on baseline EQ-5D from the “correctly” specified model (the ANCOVA model) multiplied by the coefficient from a regression of the omitted variable (baseline EQ-5D) on the included variable (treatment) [8]. The coefficient on baseline EQ-5D from the ANCOVA model ($\beta_{j0})$ is unknown for the aggregate data studies. This coefficient is therefore assumed to be exchangeable with the coefficients obtained from the studies for which individual patient data was available and can therefore be estimated as:

| (E3) |
| --- |

$$\beta_{\mathrm{pred}}\sim N(\beta_{0},\sigma_{\beta_{0}})$$

$\beta_{0}$and $\sigma_{\beta_{0}}$are estimated by assuming that the coefficient on the baseline outcome measure for each individual patient data trial ($\beta_{j0}$) is drawn from a normal distribution with mean $\beta_{0}$ and variance $\sigma_{\beta_{0}}$. The covariate from the regression of baseline EQ-5D on treatment is simply the difference in baseline EQ-5D between treatment and control for the comparison of interest (i.e. $Y_{kbj0}$).

The estimated study-specific treatment effects ($\delta_{\mathrm{kbj}}$) from the individual patient and aggregate data inform a common random effects consistency model:

| (E4) |
| --- |

$$\delta_{\mathrm{kbj}}\sim N\left( d_{\mathrm{kb}},\sigma^{2} \right)$$

$$d_{\mathrm{kb}}=d_{k1}-d_{1b}$$

where $d_{\mathrm{kb}}$ represent the treatment effects for the comparison of treatment *k* to treatment *b*, these are assumed to be derived from a set of basic parameters ($d_{k1}$) which estimate the treatment effect of treatment k relative to the reference treatment (k=1) where $d_{11}$=0.

Priors were defined to be vague and were specified as follows: $\alpha_{\mathrm{bj}}\sim N(0,{10}^{4})$; $d_{k1}\sim N(0,{10}^{3})$; $\sigma_{k}\sim Unif(0,2)$; $\beta_{0}\sim N(0,{10}^{6})$; $\sigma_{\beta_{0}}\sim Unif\left( 0,2 \right); \sigma\sim Unif(0,2)$. Vague priors were used to allow the trial data to “speak for itself”. An alternative would have been to incorporate informative priors reflecting knowledge of: the effectiveness of the interventions in other indications, their mechanisms of action or similarity between interventions. Although informative priors could have been elicited from relevant experts[9], the presence of divergent views regarding the interventions appraised here would have made obtaining representative estimates challenging.

Correlation in the random effects from trials with three or more arms were accounted for using previously published methods.[10] Where standard errors were not reported and could not be derived from the reported data, they were imputed using the methods described by Dakin *et al* which allow for the uncertainty in the imputation process.[11] If outcome data were missing for patients within the individual patient data these cases were dropped from the analysis (this occurred in 21% of all cases). If baseline data were missing for the aggregate data studies, the difference at baseline ($Y_{kbj0}$) was assumed to be zero.

Convergence was assessed by inspecting trace plots, density plots and Brooks-Rubin-Gelman plots as well as autocorrelation plots.[12] Efficiency was assessed by comparing Monte Carlo error to the posterior standard deviation. All models were run for 100,000 “burn in” iterations and a further 100,000 samples. Model fit was assessed using the posterior residual deviance and the deviance information criteria. Inconsistency was assessed using the methods set out in Dias *et al* [13] whereby the fit of the consistency model is compared to the fit of an inconsistency model. As recommended in Dias *et al* [13] changes in the model fit associated with using the inconsistency model were assessed using “omnibus” diagnostics (DIC; posterior mean residual deviance). Residual deviance contributions for each data point were also reviewed for both consistency and inconsistency models.[13]

The network meta-analysis was undertaken in WinBUGs version 1.4.3[14] called via R2WinBUGS in the statistical software R[15].

# 4. Networks of evidence

**(a) All trials**

**(b) Trials at low risk of selection bias**

**Fig S1: Network of evidence.** Points represent comparators and lines describe treatment comparisons for which direct trial evidence is available. The line’s thickness is proportional to the number of studies informing a comparison, thus, the thicker the line the larger the number of trials available for that comparison.

**Supplementary file references**

1. Rowen D, Brazier J, Roberts J. Mapping SF-36 onto the EQ-5D index: how reliable is the relationship? *Health Qual Life Outcomes*. 2009;7:27. Epub 2009/04/02. doi: 1477-7525-7-27 [pii]

10.1186/1477-7525-7-27. PubMed PMID: 19335878; PubMed Central PMCID: PMC2683169.

2. Maund E, Craig D, Suekarran S, Neilson A, Wright K, Brealey S, et al. Management of frozen shoulder: a systematic review and cost-effectiveness analysis. *Health Technol Assess*. 2012;16(11):1-264. Epub 2012/03/13. doi: 10.3310/hta16110. PubMed PMID: 22405512.

3. Gray AM, Rivero-Arias O, Clarke PM. Estimating the association between SF-12 responses and EQ-5D utility values by response mapping. *Med Decis Making*. 2006;26(1):18-29. Epub 2006/02/24. doi: 26/1/18 [pii]

10.1177/0272989X05284108. PubMed PMID: 16495197.

4. Barton GR, Sach TH, Jenkinson C, Avery AJ, Doherty M, Muir KR. Do estimates of cost-utility based on the EQ-5D differ from those based on the mapping of utility scores? *Health Qual Life Outcomes*. 2008;6:51. Epub 2008/07/16. doi: 1477-7525-6-51 [pii]

10.1186/1477-7525-6-51. PubMed PMID: 18625052; PubMed Central PMCID: PMC2490675.

5. Gu NY, Bell C, Botteman MF, Ji X, Carter JA, van Hout B. Estimating preference-based EQ-5D health state utilities or item responses from neuropathic pain scores. The patient. 2012;5(3):185-97. doi: 10.2165/11630970-000000000-00000. PubMed PMID: 22765255.

6. Chan KK, Willan AR, Gupta M, Pullenayegum E. Underestimation of Uncertainties in Health Utilities Derived from Mapping Algorithms Involving Health-Related Quality-of-Life Measures Statistical Explanations and Potential Remedies. *Med Decis Making*. 2014:0272989X13517750.

7. National Institute for Health and Care Excellence. Osteoarthritis: Care and management in adults. London: NICE; 2014.

8. Wooldridge J. Introductory econometrics: A modern approach: Cengage Learning; 2012.

9. Soares MO, Bojke L, Dumville J, Iglesias C, Cullum N, Claxton K. Methods to elicit experts’ beliefs over uncertain quantities: application to a cost effectiveness transition model of negative pressure wound therapy for severe pressure ulceration. Statistics in medicine. 2011;30(19):2363-80.

10. Dias S, Sutton AJ, Ades AE, Welton NJ. Evidence synthesis for decision making 2: a generalized linear modeling framework for pairwise and network meta-analysis of randomized controlled trials. Med Decis Making. 2013;33(5):607-17. doi: 10.1177/0272989X12458724. PubMed PMID: 23104435; PubMed Central PMCID: PMC3704203.

11. Dakin H. Review of studies mapping from quality of life or clinical measures to EQ-5D: an online database. *Health Qual Life Outcomes*. 2013;11:151. doi: 10.1186/1477-7525-11-151. PubMed PMID: 24010873; PubMed Central PMCID: PMC3844400.

12. Brooks SP, Gelman A. General Methods for Monitoring Convergence of Iterative Simulations. *J Comput Graph Stat*. 1998;7(4):434–55.

13. Dias S, Welton NJ, Sutton AJ, Caldwell DM, Lu G, Ades AE. Evidence synthesis for decision making 4: inconsistency in networks of evidence based on randomized controlled trials. *Med Decis Making*. 2013;33(5):641-56. doi: 10.1177/0272989X12455847. PubMed PMID: 23804508; PubMed Central PMCID: PMC3704208.

14. Lunn DJ, Thomas A, Best N, Spiegelhalter D. WinBUGS - A Bayesian modelling framework: concepts, structure, and extensibility. *Stat Comput*. 2000;10(4):325-37.

15. Sturtz S, Ligges U, Gelman A. R2WinBUGS: A Package for Running WinBUGS from R. *J Stat Softw*. 2005;12(3).
